# Barrier–Immune Gating Failure in Gut–Brain Neuroinflammation: A Working Hypothesis and Conceptual Framework

**DOI:** 10.1155/bn/6222269

**Published:** 2026-08-20

**Authors:** Jiabao Liao, Yide Chen, Yanming Lu, Fei Qu, Xuehua Xie, Zhixia Yang, Weibo Wen

**Affiliations:** ^1^ Jiaxing Hospital of Traditional Chinese Medicine, Jiaxing, Zhejiang, China; ^2^ Yunnan University of Chinese Medicine, Kunming, Yunnan, China, ynutcm.edu.cn; ^3^ Kunming Medical University, Kunming, Yunnan, China, kmmc.cn

**Keywords:** barrier–immune coupling, boundary-associated macrophages, disease-associated microglia, fgut–brain axis, meningeal lymphatics, neuroinflammation, skull bone marrow–meningeal axis, transcortical vascular channels

## Abstract

In the past two decades, advances in microbiomics and neuroimmunology have reframed the concept of “central immune privilege.” Rather than simply proving a gut–brain connection, the translational challenge now lies in identifying how gut‐derived signals are transmitted, filtered, amplified, or buffered across different neural, immune, metabolic, vascular, and barrier‐related routes. Based on a structured narrative synthesis of experimental, translational, and clinical literature, this review presents barrier–immune‐coupled gating failure as a working hypothesis and conceptual framework, rather than as a unifying explanation for all gut–brain interactions. Within this framework, three analytically separable but interacting dimensions are emphasized: (1) entry gating, referring to the threshold at which peripheral signals influence CNS boundary states; (2) boundary translation, in which signals are filtered or reshaped at interfaces such as the blood–brain barrier (BBB), blood–cerebrospinal fluid barrier (BCSFB), and meningeal immune zones; and (3) clearance gating, involving CSF drainage, meningeal lymphatics, glymphatic exchange, and protein or inflammatory‐signal clearance. We highlight how gut‐derived signals—filtered by the intestinal mucosa and gut‐associated lymphoid tissue (GALT)—interact with immune set points and structural barriers. These interactions are modulated by factors such as short‐chain fatty acids (SCFAs) and metabolite load, influencing whether signals are buffered or amplified. Recent findings show that skull bone marrow can directly supply immune cells to the meninges via transcortical vascular channels (TCVs), forming a responsive border niche susceptible to intestinal inflammation. At the cellular level, the review focuses on boundary‐associated macrophages (BAMs) and disease‐associated microglia (DAM) states, which bridge peripheral disruptions and central neuroinflammation. In selected contexts, reduced boundary buffering or impaired clearance may increase the likelihood that interface inflammation extends toward the parenchyma, particularly in proteinopathies with persistent inflammatory or protein burden. Finally, we propose a biomarker matrix for mechanistic stratification, which may help prioritize hypothesis‐guided evaluation of barrier repair, drainage enhancement, immune set‐point modulation, and cell‐state reprogramming, while acknowledging that current human intervention evidence remains modest, inconsistent, or context‐specific. By defining its scope and testable predictions, this framework may help move selected gut–brain studies from broad association toward more stratified and mechanism‐oriented investigation.

## 1. Introduction

“Central immune privilege” is not synonymous with “isolation from the periphery.” Strict control of molecular and cellular flow through the multilayered border structure is essential for the integrity of the central nervous system [1]. These boundaries are divided into three categories by contemporary neuropathology: the meningeal barrier with its immunological interface, the blood–brain barrier (BBB), and the blood–cerebrospinal fluid barrier (BCSFB), which is centered on the choroid plexus [2]. Peripheral signal accessibility, presentation, and response intensity are influenced by these structures. Therefore, it is not necessary for the gut microbiota to “enter the brain” to influence the central nervous system; instead, immunological signals and enterogenous metabolism can change the status of border cells, which in turn affects the stability of the central microenvironment [3]. For instance, short‐chain fatty acids (SCFAs) promote barrier homeostasis and tight junction phenotypes. However, boundary cells may be stressed by an ongoing high concentration of proinflammatory cytokines, which can alter permeability, transport, and receptor expression [4, 5]. Multicellular interactions involving elements such as the endothelium, pericytes, perivascular fibroblast‐like cells, and choroid plexus epithelium frequently lead to such alterations [6]. Recent anatomical discoveries have clarified the structural mechanisms through which peripheral signals are processed in the body. In addition to systemic circulation, myeloid cells can swiftly cross the meningeal border owing to the near‐field immune supply channel that runs between the meninges and bone marrow of the skull. This implies that bone marrow “nearby mobilization” may also impact the meningeal response threshold and inflammation persistence, indicating that border immunity is not merely a passive reaction to systemic inflammation [7–9]. However, clinical investigations have demonstrated that gains in key clinical objectives are not always correlated with changes in specific biomarker levels. Therapeutic effects may be constrained by variables such as disease stage, heterogeneity, and intervention window. It will be difficult to explain these disparities if gut–immune–brain research remains at the “association‐based catalogue” level [10, 11]. To avoid another association‐based narrative, this review uses barrier–immune gating failure as a working hypothesis for organizing selected evidence at CNS interfaces, with key readouts summarized in Table 1 and Tables S1 and S2. This framework is not intended as a single linear or universal gut–brain pathway because neural, autonomic, metabolic, neuroendocrine, immune, vascular, barrier, and clearance routes may operate in parallel. Its intended scope, limitations, and evidence requirements are summarized in Box S1.

**Table 1:** Evidence tiering and minimal readouts for key gating nodes.

Module	Dominant evidence (human/animal/in vitro)	Minimal readouts	Translational levers (stratification/intervention)
TCVs/calvarial bone marrow–meninges axis (bone marrow priming)	Human: mostly correlational/proxy indicators; animal: trackable migration and temporality; in vitro/ex vivo: lineage bias and chemotaxis/adhesion axes	TCV traffic volume (tracing/spatial); meningeal myeloid composition; coupled blood/CSF inflammation–hematopoiesis readouts	Stratify by “bone marrow priming + meningeal reactivity”; target chemotaxis/adhesion axes or repair upstream barriers [12]
mLVs vs. glymphatic system (clearance gating)	Human: imaging/fluid markers suggest clearance efficiency; animal: causal tests via manipulating mLVs/AQP4; in vitro: mechanisms in lymphatic endothelium and astrocytic endfeet	Tracer‐based clearance kinetics; AQP4 polarity; CSF turnover/drainage indices; PVS structural readouts	Align endpoints to “clearance rate”; enhance drainage and improve sleep and vascular compliance [13, 14]
BAMs (PVS gatekeepers: TMAO–CAA)	Human: metabolites/imaging correlate with CAA/microbleeds; animal: validate metabolic input → BAM function → deposition; in vitro: lipid stress/lysosomal pathways	BAM state signatures; PVS clearance readouts; imaging endpoints of CAA/microbleeds; TMAO/choline metabolism profiles	Stratify by metabolic load + perivascular readouts; target metabolic pathways or boost perivascular clearance [15–17]
SCFAs (HDAC/threshold tuning)	Human: mostly correlational; animal: supplementation/depletion reveals threshold shifts; in vitro: HDAC/acetylation with cell‐type‐specific effects	SCFA levels; microglial homeostatic genes and response curves; barrier/IgA readouts	Align endpoints to “threshold readouts”; use prebiotics/postbiotics as upstream strategies [18–20]
Tryptophan–AhR axis (background set‐point)	Human: metabolomic profiles associate with phenotypes; animal: AhR perturbation tests persistence; in vitro: transcriptional programs in astrocytes/border cells	AhR target‐gene signatures; cytokine background in border cells; glial persistence readouts	Stratify by “AhR signature + border background”; target metabolic shunting or receptor pathways [21, 22]
DAM/BAM state trajectories (replacing M1/M2)	Human: single‐cell/spatial alignment to lesions; animal: module interventions validate across stages; in vitro: coupling of lipid handling–lysosome–inflammation	Trajectory stage markers; spatial localization (meninges/PVS/parenchyma); phagolysosomal endpoints	Stratify by “trajectory stage + spatial niche”; target lipid handling/lysosomal modules [23, 24]
Live biotherapeutic interventions (LBT)	Human: heterogeneous efficacy and endpoints; animal: mechanistic insight and safety windows; in vitro: strain functions and metabolic outputs	Strain functional outputs; barrier/immune set‐point; border readouts (CSF/imaging)	Stratify by “barrier/immune set‐point”; prioritize postbiotics or defined consortia [25–27]

Abbreviations: BAMs, border‐associated macrophages; CAA, cerebral amyloid angiopathy; DAM, disease‐associated microglia; FMT, fecal microbiota transplantation; mLVs, meningeal lymphatic vessels; PVS, perivascular spaces; SCFAs, short‐chain fatty acids; TCVs, transcortical vascular channels; VNS, vagus nerve stimulation.

### 1.1. Review Strategy and Evidence Selection

This article was designed as a narrative review rather than a systematic review or meta‐analysis. To improve transparency, we performed a structured literature search in PubMed, Web of Science, Scopus, and Google Scholar for studies published between January 2000 and January 2026, with the final search updated in January 2026. Search terms included combinations of “gut–brain axis,” “microbiota–gut–brain axis,” “neuroinflammation,” “blood–brain barrier,” “blood–cerebrospinal fluid barrier,” “meningeal lymphatics,” “glymphatic system,” “skull bone marrow,” “transcortical vascular channels,” “boundary‐associated macrophages,” “disease‐associated microglia,” “short‐chain fatty acids,” “tryptophan metabolism,” “bile acids,” “fecal microbiota transplantation,” “vagus nerve stimulation,” “Alzheimer’s disease,” and “multiple sclerosis.” Studies were prioritized when they provided mechanistic or translational evidence on gut‐derived signals, CNS barrier regulation, meningeal immunity, immune‐cell trafficking, glial state transitions, clearance pathways, or microbiota‐related interventions. Purely correlative studies without CNS‐relevant mechanistic or clinical endpoints, duplicate reports, and articles outside the scope of gut–immune–brain communication were not emphasized. Because the evidence base includes animal experiments, human observational studies, clinical trials, and in vitro or ex vivo models, studies were evaluated according to mechanistic strength, anatomical localization, cell‐type specificity, temporal information, intervention reversibility, and translational relevance rather than by formal risk‐of‐bias scoring. To reduce selective interpretation, we also considered negative, inconsistent, and nonreplicated findings, especially when they challenged microbiota‐directed interventions, probiotic strain specificity, FMT efficacy, vagus nerve stimulation (VNS) generalizability, or the causal ordering of barrier and immune events. Animal studies were not treated as direct clinical proof, human observational studies were not used alone to infer causality, and in vitro findings were considered supportive only when aligned with anatomical, temporal, or functional evidence. Accordingly, associative links between microbial metabolites, immune‐cell states, vascular pathology, and transcriptional readouts were described as candidate mechanisms unless supported by temporal ordering, perturbation experiments, intervention reversibility, or convergent human evidence.

## 2. Barriers, Boundaries, and Special Pathways: The Multidimensional Anatomical Foundation From the BBB to the Cranio–Meningeal Immune Interface

**Figure 1:**
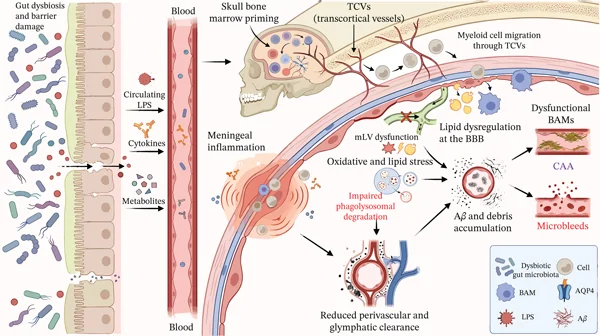
Gut‐derived entry drivers converge with border clearance failure to promote CNS vascular amyloid pathology. Gut dysbiosis and mucosal barrier damage increase the systemic flux of LPS, inflammatory cytokines (e.g., IL‐1*β* and TNF), and metabolites (e.g., TMAO). These signals prime the skull diploë bone marrow (epigenetic/metabolic reprogramming), generating primed myeloid cells that can rapidly access the meninges via transcortical vascular channels (TCVs) and amplify inflammation. In parallel, impaired meningeal lymphatic vessels (mLVs) reduce the drainage of deep cervical lymph nodes. At brain boundaries, BBB/endothelial lipid handling (e.g., CD36) and dysfunctional border‐associated macrophages (BAMs; Lyve1^+^ and CD163^+^) promote lipid overload/oxidative stress, impair phagosome‐lysosome degradation, disrupt astrocytic AQP4 polarity, and reduce perivascular and glymphatic flow. Combined “entry + clearance” failure favors A*β*/debris accumulation, accelerates cerebral amyloid angiopathy (CAA), and increases the risk of microbleeds.

This section narrows the working hypothesis to CNS boundary and clearance structures. Evidence strength differs across models: animal studies are most informative for timing and causality, human studies mainly provide stratification and disease relevance, and in vitro or ex vivo systems help resolve cell‐specific pathways [28]. The meningeal immune interface, skull bone marrow–meningeal pathway, BBB/BCSFB, and CSF–lymphatic drainage system are therefore discussed as interacting modules, not as a fixed sequence. Direct human evidence linking intestinal perturbations to these structures remains limited, so these modules should be viewed as candidate amplification sites rather than uniformly validated steps (Figure 1).

### 2.1. Cranial Bone Marrow–Meningeal Axis

In the past, “remote recruitment” from the systemic circulation was the main cause of meningeal inflammation. Nonetheless, new anatomical data suggest that the intracranial immunological interface is closely linked to the diploic bone marrow of the skull [29]. Transcortical vascular channels (TCVs) allow myeloid cells to move rapidly from the skull bone marrow to the meninges. In contrast, the mobilization threshold and cell lineage in the nearby bone marrow niche may be affected by inflammatory conditions in the meninges. The theory of “bone marrow priming,” which relates intestinal disruptions to border immunological responses, emerges within this structural framework. A weakened intestinal mucosal barrier can cause low‐grade endotoxemia and systemic inflammation, which can permanently change the metabolic and epigenetic programming of hematopoietic and myeloid cells, increasing their mobilization and heightening the meningeal response [30, 31]. This approach has several advantages, including measurable results (bone marrow activation and border inflammation), distinct upstream drivers (enterogenic low‐grade inflammation/endotoxin exposure), distinctive carriers (myeloid cells), and identifiable structural channels (TCVs). Therefore, the skull bone marrow–meningeal axis is important because it provides a precise window for observation, allowing for the monitoring of changes in TCV flux, changes in the composition of the meningeal myeloid system, and possible adjustments after the intestinal inflammatory load has been corrected.

### 2.2. Meningeal Lymphatic Vessels (mLVs) and Lymphatic System

The prompt removal of waste and inflammatory signals is as important for maintaining central homeostasis as the entry of peripheral inputs [32]. Therefore, the BBB and drainage system can be divided into two types of gates: the “entry gate,” which is the BBB, and the “clearance gate,” which is the lymphatic system and mLVs. These elements serve different functions. For example, the mLVs of the dura mater transport cerebrospinal fluid and large molecular debris to cervical lymph nodes. The glymphatic system depends on the polarized distribution of aquaporin‐4 (AQP4) in astrocytic endfeet to promote convective exchange between interstitial fluid and cerebrospinal fluid within perivascular channels. Together, meningeal lymphatics and the glymphatic system support macromolecular drainage, solute exchange, and perivascular waste clearance. Short‐term oscillations can become ongoing loads if any component of the system is damaged [33–35]. Examining the central clearance system and the functional output structure and dynamics of the microbiota within the same framework is a straightforward method from the perspective of “intestinal regulation.” A decrease in microbiota function output makes AQP4 polar localization more susceptible to disruption, potentially lowering cerebrospinal fluid turnover and convection efficiency around blood vessels, according to current evidence, mainly from microbiota deprivation/imbalance models and tissue analysis. Additionally, the drainage capacity and structure of the mLVs may be affected. An integrated “structure‐dynamic‐function” readout that includes the following factors is the most effective way to evaluate this relationship: AQP4 placement, cerebrospinal fluid turnover/tracer dynamics, mLV structure and drainage efficiency, and the degree of coupling between these indicators and SCFA output [36–39]. Therefore, a kinetic approach is necessary to address “clearance slowing down”: Which changes first, the protein load or the tracer clearance curve? Alternative reasons, such as vascular risk, sleep/rhythm, or age effects on AQP4 polarity, should be prioritized if they are not synchronized.

### 2.3. Boundary‐Associated Macrophages

Peripheral metabolites interact with boundary cells close to the vascular wall through the Virchow–Robin space (PVS), the brain’s milieu where clearance and immune surveillance meet [40]. Located in boundary regions such as the PVS, meninges, and choroid plexus, border‐associated macrophages (BAMs) are involved in barrier maintenance, clearance of debris and protein aggregates, and establishment of local immunological thresholds during homeostasis [41]. By establishing BAMs as “perivascular boundary regulators,” the connection between major pathogenic endpoints and enterogenous humoral signals is condensed into a traceable cellular node, transcending the narrative of systemic inflammation. BAMs are not a homogeneous population; markers and transcriptional traits, such as Lyve1, MHCII, and CD163, can be used to separate them into subgroups. These include subgroups that are scavenger‐like (homeostatic clearance/tissue maintenance) and antigen‐presenting‐like (immune amplification) [41, 42]. This division of work allows BAMs to be the targets of “context‐dependent remodeling” of the immune‐clearance axis border by an inflammatory backdrop and metabolic load. For example, trimethylamine N‐oxide (TMAO) stimulates oxidative stress‐related pathways and lipid absorption in macrophages. Lipid overload and oxidative stress may result from TMAO′s upregulation of lipid absorption molecules, such as CD36, in BAMs [43]. It is crucial to remember that improved intake does not always equate to improved clearance. BAM malfunctions, cell death, and local vacancies can result from phagocytic‐lysosomal degradation being hindered by chronic exposure to inflammatory signals [44]. From a structural perspective, this could lower the capacity for perivascular clearance, and reduced BAM processing efficiency for perivascular A*β* and related fragments can result in A*β* deposition along the vascular wall, accelerating the progression of cerebral amyloid angiopathy (CAA) and increasing the risk of microbleeds and cerebrovascular events [45]. Consequently, aberrant intestinal metabolite profiles and cerebrovascular protein deposits may be organized as a candidate, testable pathway rather than as an established causal chain. This pathway includes metabolite changes such as TMAO, altered BAM receptor expression or metabolic stress, impaired PVS clearance kinetics, A*β* deposition along the vascular wall, and CAA progression. However, these links should be interpreted cautiously because TMAO‐related macrophage remodeling, lipid stress, vascular injury, and amyloid deposition may also arise from broader cardiometabolic or neurodegenerative processes. Therefore, the TMAO–BAM–CAA pathway should be considered supported only when increased metabolite exposure, impaired phagocytic–lysosomal processing, and worsening vascular amyloid pathology are temporally aligned or experimentally reversible [46, 47]. The percentage and spatial distribution of BAMs subsets (PVS/meninges/choroid plexus), CD36 and lipid stress signaling axis, phagocytic and lysosomal functional indicators, PVS tracer clearance kinetics, and imaging and pathological endpoints of CAA/microbleeds are important readouts. The gut–brain link can be advanced beyond correlation to quantifiable, comparable, and falsifiable cell‐structural mechanisms using these readouts. The crucial factor is not just “BAMs being more active,” but also whether the lipid load disrupts the phagocytic–lysosomal connection. The TMAO‐CAA chain is deemed closed only when both “intake increases” and “degradation decline” occur simultaneously.

## 3. Three‐Molecule Messengers and Epigenetic Remodeling

**Figure 2:**
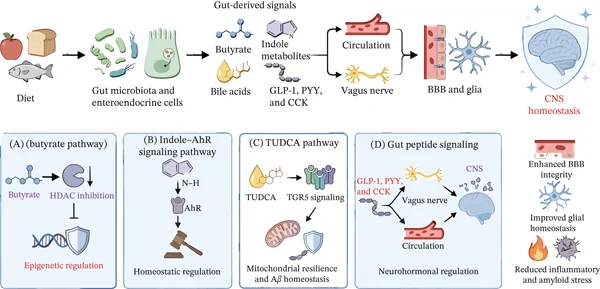
Gut‐derived messengers tune CNS immune thresholds and cellular homeostasis. Dietary substrates shape gut microbiota outputs (SCFAs, tryptophan‐indole derivatives, and secondary bile acids) and enteroendocrine gut peptides (GLP‐1, PYY, and CCK) with parallel vagal signaling. These signals reach CNS interfaces (BBB/boundaries) to set glial and barrier “thresholds” via four modules: (A) SCFAs (notably butyrate) inhibit HDACs and raise homeostatic/epigenetic reaction thresholds; (B) indole‐AhR signaling maintains an inhibitory baseline that can be lost during pathological transitions (e.g., EAE); (C) bile acids (e.g., TUDCA) acting via TGR5/FXR support mitochondrial/organelle resilience and influence A*β* stability/handling; and (D) gut peptides provide dual tuning through rapid vagal coding (brainstem) and slower hormonal background tuning.

Intestinal microbial metabolites should not be treated simply as a molecular list, but neither should they be interpreted as uniformly directional instructions to the CNS. Their effects are context‐dependent and may vary according to host diet, microbial ecology, receptor distribution, disease stage, dose, timing, and cell type. SCFAs, tryptophan metabolites, bile acids, and intestinal peptides may support barrier and immune homeostasis in some settings, but they may show weak, inconsistent, or indirect associations with CNS outcomes in others. Therefore, this section evaluates these axes by asking not only whether they fit the gating failure framework, but also where the evidence remains indirect, model‐dependent, or insufficient for causal inference. Throughout this section, metabolite–cell‐state relationships are therefore presented as hypotheses requiring temporal, perturbational, or receptor‐level validation, not as completed causal chains (Figure 2).

### 3.1. SCFAs

Acetic, propionic, and butyric acids are examples of SCFAs, which are important markers of microbiota function and are frequently used to show the continuum from “dietary substrate–microbiota metabolism–mucosal barrier–peripheral immune setpoint.” SCFAs are important for both receptor‐mediated signaling and their epigenetic impact on cellular state thresholds in relation to central immune thresholds. Butyrate, which is recognized for its histone deacetylase (HDAC)‐inhibitory activity, has been associated with increased histone acetylation markers such as H3K9ac in germ‐free or antibiotic‐treated animal models and in vitro glial systems [18–20]. In experimental systems, HDAC inhibition may be linked to more stable homeostatic transcriptional programs, but this should not be interpreted as direct evidence that peripheral SCFA changes because CNS transcriptional threshold shifts in humans [48, 49]. In terms of phenotype, this indicates that the activation amplitude and speed of proinflammatory transcriptional modules are constrained by external stimuli, whereas homeostatic microglial marker genes, such as purinergic receptor P2Y12 (P2ry12) and transmembrane protein 119 (Tmem119) are easier to maintain [50]. Hence, microglia are more vulnerable to “homeostasis instability” at the chromatin level when the metabolic production of microbiota declines, potentially entering an immature or highly reactive state. This alteration is more likely to manifest as a drift in threshold parameters, changing the starting point, slope, or peak of the transcriptional response curve rather than directly translating into changes in inflammatory factors [51, 52]. According to available research, it is more consistent to think of SCFAs as “threshold regulators” rather than “necessarily anti‐inflammatory.” Therefore, rather than correlating receptor expression or peripheral concentration, evidence should focus on threshold reset indications, such as acetylation profiles, chromatin immunoprecipitation (ChIP) signals, chromatin accessibility, persistence of homeostatic programs, and poststimulation transcriptional dynamics. Therefore, SCFA‐related associations should be treated as evidence of possible threshold modulation only when metabolite changes are temporally aligned with receptor engagement, acetylation profiles, chromatin accessibility, persistence of homeostatic programs, or poststimulation transcriptional dynamics.

### 3.2. Tryptophan Metabolism and Aryl Hydrocarbon Receptor (AhR) Signaling

Tryptophan metabolism is important for research because it offers a traceable framework for the dispersion of intestinal ecological signals, unlike its wide variety of products. Tryptophan experiences quantifiable alterations in the kynurenine, microbial indole derivative, and 5‐HT‐related pathways in response to different inflammatory stressors [53]. Emphasis is placed on the indole‐AhR axis, which successfully connects “intestinal output → brain receptor → glial program.” The AhR binds to indole derivatives from various microbiological sources. AhR is not just “anti‐inflammatory/proinflammatory” in astrocytes, it also acts as a threshold regulator at the transcriptional level. The main effect of AhR activation is a reset in transcriptional control, which raises the inflammatory threshold at the tissue level by limiting the expression of proinflammatory modules linked to NF‐*κ*B and making it harder to initiate and maintain inflammatory genes by enlisting inhibitory complexes and coinhibitory factors [54, 55]. According to this theory, the “inhibitory baseline” of the local microenvironment is the first alteration in AhR signaling in astrocytes. In response to peripheral input or local damage signals, this lessens the possibility that microglia will cross the threshold and join a self‐sustaining proinflammatory circuit without generating an instantaneous unidirectional shift in a particular inflammatory factor [56]. Experimental autoimmune encephalomyelitis (EAE), a commonly used model of MS, provides a pathological context in which this pathway can be tested: astrocytes′ capacity to reduce CNS inflammation is reduced when AhR signaling is lacking or compromised, making demyelination and inflammatory dissemination more difficult to manage [57–59]. Consequently, the following is a more accurate statement: the main alteration in the course of the disease is not a “sudden increase in proinflammation” of microglia, but rather a deterioration of the astrocyte‐provided inhibitory backdrop, which allows inflammation to pass beyond the threshold and continue to progress. Assuming “AhR as the transcriptional threshold regulator,” the minimum testable timing criterion should be a decrease in astrocyte inhibitory transcription baseline (or associated epigenetic/chromatin readout changes), followed by an increased likelihood of microglia crossing the threshold and increased persistence of inflammation. A reexamination of the causal direction and other explanations is necessary if the opposite order is observed, as this axis may be more of a contemporaneous response than an upstream cause.

### 3.3. Bile Acids and Mitochondrial Homeostasis

Microorganisms alter primary bile acids, affecting their composition and biological activity, making them key components of gut–liver cometabolism. Bile acid signaling is important in the neurological system not only because it reflects metabolic backgrounds but also because it can directly communicate “intestinal metabolic changes” to mitochondrial homeostasis at the organelle level, influencing tissue susceptibility and cell fate [60]. For example, in certain models and pathological situations, bile acid derivatives such as tauroursodeoxycholic acid (TUDCA) have demonstrated protective benefits in central cells. Through bile acid receptor‐related pathways like FXR/TGR5 and its downstream signals, these chemicals mechanistically control the mitochondrial function of neurons and glial cells. This modulation results in reduced oxidative stress, decreased apoptosis, and a more stable mitochondrial membrane potential (*ΔΨ*m) [61–63]. In contrast to the more general question of “anti‐inflammatory effects,” this method offers a more specific causal mediator: whether energy homeostasis is preserved and whether cells are less prone to stress, damage, and death. As a result, the bile acid axis is positioned in this chapter as an “entry point of organelle mechanism,” and the evidence is arranged according to indicators that are directly related to mitochondrial homeostasis, such as *ΔΨ*m, ROS/oxidative stress markers, mitochondrial dynamics/quality control markers, and apoptotic pathway markers. These organelle‐level readouts should be matched with tissue‐level outcomes, such as imaging, pathological damage indicators, and behavioral phenotypes, to prevent the overinterpretation of correlations as causality. Evidence containing temporal information or intervention reversibility should be prioritized in future studies to address these limitations of the current study.

### 3.4. Intestinal‐Derived Peptides and Gut–Brain Neuroendocrine Signaling

Peptide hormones released by intestinal endocrine cells, notably glucagon‐like peptide‐1 (GLP‐1), peptide YY (PYY), and cholecystokinin (CCK), function closer to the signal layer and provide high temporal resolution, in contrast to metabolite‐driven sluggish reprogramming [64]. After eating and nutrient perception, these hormones rapidly alter the activity patterns of vagal afferents and brainstem integration circuits. This rapid change influences the vulnerability to neuroinflammation and controls the central responses to peripheral input. Current evidence suggests two complementary modes of gut–brain neuroendocrine signaling. The first, referred to here as the neural coding pathway, describes the rapid transmission of nutrient‐ and peptide‐related information through vagal afferents and brainstem circuits over minutes to hours. In this mode, intestinal peptides alter vagal firing patterns and autonomic output, thereby influencing central state regulation through brainstem and hypothalamic networks. The second, referred to here as the background shaping pathway, describes slower modulation of the inflammatory and metabolic context in which glial and immune cells respond. In this mode, peptide‐related metabolic improvement or reduced peripheral inflammatory tone may indirectly alter glial responsiveness and central susceptibility over longer time scales [65, 66]. For instance, it is important to avoid directly attributing any central benefits to “neuroimmunosuppression” when discussing GLP‐1. Presenting the “indirect effects of metabolic improvement” in addition to the “direct effects mediated by receptors,” and substantiating these attributions with a distinct endpoint system is a more trustworthy method. In addition to intermediate measures of inflammation, glial health, and neural circuit activity in the brainstem, meninges, and brain parenchyma, this entails reporting metabolic outcomes such as body weight and insulin sensitivity. The contributions of the two pathways can be separated through tissue‐specific manipulation, receptor blockage, or time‐window segmentation [67]. Therefore, intestinal‐derived peptides are best viewed as rapid neuroendocrine modulators that interact with slower metabolite‐dependent processes. Their relevance to the proposed framework lies in their potential to influence short‐term neural gain and longer‐term inflammatory or metabolic susceptibility, rather than in a single direct neuroimmune pathway.

## 4. Immune Cell Migration and Phenotypic Remodeling: From Peripheral Education to Central Residency

**Figure 3:**
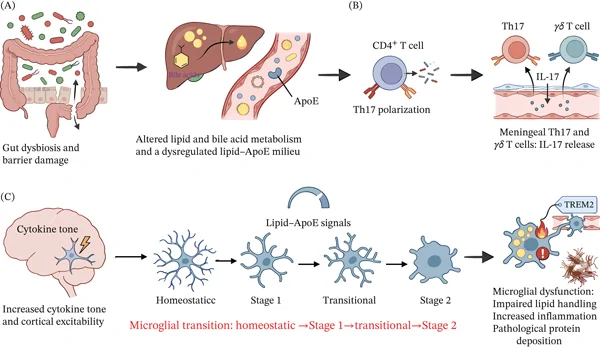
Gut lipid‐immune priming at the meningeal border shapes microglial state transition and protein pathology. (A) Barrier damage and dysbiotic microbiota remodel lipid/bile acid metabolism, creating an altered lipid‐ApoE milieu that promotes Th17 polarization. (B) Th17/*γδ* T‐cells in the meninges release cytokines (notably IL‐17), sustaining a cytokine baseline and increasing cortical excitability/synaptic plasticity changes. (C) Microglia progress from a homeostatic state to Stage 1 homeostatic withdrawal (TREM2‐independent; ↓P2ry12/Cx3cr1/Tmem119) and a plastic/transitional state, the threshold of which is tuned by gut lipid/ApoE signals. Stage 2 is either effective (TREM2‐dependent; ↑ApoE/Lpl/Cst7/Clec7a/Itgax) with an organized barrier‐like sealing response or impaired (“stage stagnation”) with ineffective lipid handling/phagocytosis, persistent inflammatory hot zones, and protein deposition/pathology.

Despite being the most reliable “transport layer” in the gut–immune–brain axis, immune cells are frequently misinterpreted. Spatial and single‐cell maps of humans show “who is where,” whereas blocking and tracking tests on animals show “who caused what.” Chemotaxis, adhesion, and metabolic stress can be distinguished using in vitro systems [68]. This chapter focuses on two crucial entry points: the background of meningeal T cells, which creates a stable cytokine baseline at the boundary, and the state trajectory of microglia, which affects the reorganization of phagocytosis, lipid processing, and inflammatory transcription programs. Using this method, evidence from various levels is not confused with comparable causality (Figure 3).

### 4.1. Microglia DAM/MGnD

Microglial phenotypes cannot be adequately summarized using the conventional “M1/M2” dichotomy. Rather, conditions associated with protein deposition and neurodegeneration are better categorized into two lineages: the microglial neurodegenerative phenotype (MGnD) and disease‐associated microglia (DAM) states. These categories represent distinct emphases on the same state trajectory across different disease situations, although they are not mutually exclusive in nature. Steady‐state monitoring programs were consistently downregulated, whereas phagocytosis, lipid metabolism, and antigen presentation were upregulated. [69]. Therefore, rather than using discrete labels, it is more acceptable to consider these states as a continuous spectrum. A “two‐stage model” is frequently used to describe this trajectory. Downregulation of Tmem119, Cx3cr1, and P2ry12 are examples of steady‐state markers that decrease in Stage 1, which is triggering receptor expressed on myeloid cells 2 (TREM2)‐independent. As the cell withdraws from homeostatic support and enters a more sensitive, malleable state responsive to local microenvironmental signals, the key change here should not be summed up as “more proinflammatory” [70]. The initiation of phagocytosis and lipid processing, along with the overexpression of Apoe, Lpl, Cst7, Clec7a, and Itgax/CD11c, are characteristics of Stage 2 (TREM2‐dependent) microglia. This stage, which is closer to the lesion′s executive layer, determines whether the lesion continues to be an active inflammatory area or creates an ordered phagocytic and sequestration structure [71]. Intestinal variables are more likely to affect the cross‐stage threshold of this trajectory within the “gate failure” framework than to determine admission into the DAM. This leads to a cautious, testable hypothesis: intestinal signaling may modulate the probability or timing of microglial transition from Stage 1 to Stage 2 by altering lipid‐substrate availability and the apolipoprotein E (APOE)‐related metabolic milieu. However, current evidence does not establish that microbiota‐derived metabolites directly drive DAM/MGnD transitions in humans. These associations should be interpreted as candidate modulatory links unless supported by temporal sequencing, cell‐type‐specific manipulation, or reversal after targeted intervention. Microglia may more easily reach the “steady‐state retreat” window of Stage 1 in the presence of dysbiosis and an elevated metabolic load, but they will find it difficult to complete the efficient phagocytic–lysosomal program and lipid processing needed for Stage 2 [72]. This is more consistent with “stalled DAM/MGnD transition,” where a persistent inflammatory zone is more likely to develop around the lesion rather than a barrier structure with sealing ability, than with “increased DAM.” Accordingly, stratification should focus on three features: the downregulation of Stage 1 homeostatic genes, including P2ry12, Cx3cr1, and Tmem119; the activation of the Stage 2 TREM2‐dependent module, including Apoe, Lpl, Cst7, Clec7a, and Itgax; and the spatial organization of the lesion microenvironment, ranging from organized barrier‐like structures to diffuse inflammatory hotspots. As a result, stratification should concentrate on determining whether the DAM/MGnD transition is stalled because the lesion area is more likely to remain an active inflammatory zone when the homeostatic program continues to deteriorate and phagocytic–lysosomal, lipid‐processing, and lesion‐containment modules are delayed.

### 4.2. Meningeal T cells

T cells in the meninges provide a long‐lasting immunological barrier against infections. On the meningeal side, cytokines from CD4^+^ and *γδ* T cells provide a local milieu that affects synaptic plasticity and neuronal excitability. [73]. Consequently, the meninges function as both flexible immune‐neural functional bridges and as immunological barriers. A distinct pathogenic pathway for this interaction is provided by a peripheral Th17 bias. The Th17 program may be permanently heightened in situations involving intestinal inflammation and barrier degradation. Thus, a local cytokine environment, mainly composed of IL‐17, is produced in the meninges as a result of some Th17 cells migrating and accumulating there [74, 75]. Quantifiable neurophysiological outcomes were correlated with the environment. Models such as MS/EAE use the correlation between the IL‐17‐related background and cortical neuron overexcitation and circuit gain upregulation to clarify the “boundary immunity–cortical excitability–behavioral/functional phenotype” chain [76]. More important than “whether it enters the brain parenchyma,” the question is whether a long‐lasting cytokine microenvironment develops on the meningeal side and whether it has the power to change excitability and plasticity [77]. Important verifiable points include the following: (1) Cell lineage: meningeal CD4^+^ and *γδ* T cell composition, kinetics, and spatial localization; (2) effector molecules: the specific functions of IL‐4, IL‐17, and IFN‐*γ* in the border backdrop; and 3) homing and localization: verifiable associations between cortical excitability markers, local cytokine gradients, and meningeal immune composition. The criterion here is not just “the presence or absence of T cells,” but also whether the meningeal side develops a sustained cytokine background and whether this background correlates with cortical excitability over time.

## 5. The Gut–Brain Axis in the Disease Spectrum

**Figure 4:**
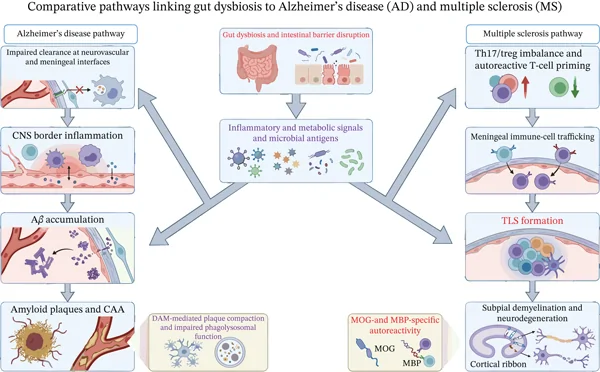
Divergent gut–brain readouts in AD and MS. Gut dysbiosis and epithelial leakage increase systemic inflammatory/metabolic signals (e.g., LPS, cytokines, and altered lipids) and gut‐derived metabolites/antigens but yield disease‐specific CNS outputs. AD: Clearance‐gating failure at the neurovascular‐meningeal interface (glymphatic/AQP4‐dependent flow and meningeal lymphatic drainage) sustains border inflammation, promoting net A*β* accumulation with plaques/CAA; DAM responses may compact plaques, whereas impaired phagolysosomal function increases neurotoxicity. MS: Gut mucosal immune dysregulation skews Th17/Treg balance and supports antigen‐driven autoreactive T‐cell priming (including MOG/MBP‐related targets), enabling meningeal trafficking, TLS formation, and subpial cortical demyelination/neurodegeneration.

The following disease examples are not intended to imply that a single gating hierarchy explains neuroinflammation across disorders. Rather, AD and MS are used as illustrative contexts in which boundary regulation, clearance failure, meningeal immune organization, or glial‐state remodeling has relatively strong evidence and can be mapped onto the proposed working model. Other neurological and psychiatric disorders may involve gut–brain communication through different dominant routes, including neural, endocrine, metabolic, vascular, or systemic immune mechanisms, and are therefore discussed more cautiously (Figure 4). Importantly, in AD and MS, gut‐related factors are discussed as modulatory influences on disease progression, inflammatory tone, barrier function, or treatment responsiveness, rather than as primary determinants of disease initiation.

### 5.1. Alzheimer′s Disease

In AD, the gating failure framework is best interpreted as a modulatory model superimposed on primary central pathological processes, including A*β* accumulation, tau pathology, synaptic dysfunction, neurodegeneration, and vascular injury. Peripheral inflammation and metabolic disorders do not directly control A*β* production, but they may influence clearance efficiency, boundary inflammation, and tissue responses after central pathology has emerged [78]. Reduced meningeal lymphatic artery‐draining capacity and poor lymphatic convective exchange are associated with microbiota dysbiosis, which can also increase the levels of circulatory inflammatory mediators and metabolic disorders. A*β* transboundary transport and interstitial clearance are hampered by this impairment, which may be related to alterations in AQP4 polarity or diminished vascular pulsation drive. A*β* is more likely to accumulate in the brain parenchyma and along the arterial walls as clearance efficiency declines, leading to CAA. A positive feedback loop focused on “boundary inflammation–clearance damage” is created when inflammation at the perivascular and meningeal interfaces further impairs drainage and convective exchange [79, 80]. Plaque‐related neurotoxicity depends on microglial reactions to plaques and the quantity of plaque deposition. By creating a surrounding plaque response and encouraging plaque compaction, a strong DAM program can reduce the exposure of surrounding tissues to soluble A*β* oligomers and other hazardous species. Periplaque response efficiency declines when phagocytic, lysosomal, and lipid processing modules are compromised, resulting in looser plaque formation with greater local inflammatory signals and neurotoxic loads [81]. Through their effects on lipid processing and APOE‐related pathways, microbiota output and host metabolic substrate bias may affect the effectiveness of the microglial transition from homeostasis to DAM and the periplaque response [82, 83]. Therefore, rather than dictating the presence of A*β* deposition, intestinal variables in AD are more likely to affect tissue response modes and disease progression rates following deposition by controlling boundary drainage/lymphoid exchange and microglial effector programs. This interpretation should be balanced against alternative explanations. A*β* accumulation and CAA can arise from aging, APOE genotype, vascular dysfunction, impaired arterial pulsatility, sleep disruption, and primary neurodegenerative processes independent of gut‐derived signals. Human microbiota studies in AD are often cross‐sectional and cannot determine whether dysbiosis precedes neurodegeneration or reflects dietary change, medication exposure, frailty, or disease stage. Therefore, in AD, the gating failure model is most persuasive when gut‐related changes are temporally linked to clearance impairment, boundary inflammation, or microglial‐state remodeling, and should be interpreted as a modifier of disease progression rather than as a primary cause of AD pathology.

### 5.2. Multiple Sclerosis

In MS, gut‐related immune signals should be interpreted within the broader context of established autoimmune mechanisms, including genetic susceptibility, EBV‐related immune priming, peripheral antigen presentation, CNS antigen specificity, and compartmentalized CNS inflammation. Changes in the intestinal immune milieu may modulate the Th17/Treg balance and influence the persistence or amplification of autoreactive T‐cell responses, but they should not be treated as primary determinants of MS initiation. The development of tertiary lymphoid structures (TLSs) on the meningeal side is an important pathogenic connection with improved spatial resolution [84–86]. TLS are more strongly associated with subcortical chronic demyelination and increasing damage and function as structured microenvironments for local antigen presentation and B‐cell clone proliferation. Peripheral immune start events can therefore be mechanically aligned at well‐defined anatomical boundaries. [86]. A possible intestinal trigger mechanism has been suggested by molecular simulations: cross‐reactive T cells may be induced by the sequence or structural similarity between intestinal bacterial antigens and myelin antigens (MOG/MBP), decreasing the threshold for autoimmune reactions [87]. In this context, meningeal T‐cell and B‐cell responses may contribute to cortical injury when they are maintained within TLS, but such effects should be interpreted alongside established CNS‐compartmentalized immune mechanisms [88]. However, MS also illustrates why the gating model must be compared with established autoimmune mechanisms. Genetic susceptibility, EBV‐related immune priming, peripheral antigen presentation, CNS antigen specificity, and treatment effects may independently shape disease activity. Microbiota‐associated Th17/Treg imbalance should therefore be interpreted as one modulatory layer rather than as a primary causal trigger in all patients. Stronger support would require longitudinal sampling before relapse, tissue‐level validation, and evidence that microbiota‐directed interventions alter meningeal immune organization or cortical injury.

### 5.3. Other Diseases

To avoid overextending the framework into a list of weak associations, this review focuses on two disease contexts with comparatively traceable evidence chains: AD and MS on two distinct chains of evidence that can be conclusively examined at various levels: MS, which considers meningeal adaptive immunity as a process readout linked to TLS and the progression of cortical damage, and AD, which considers clearance/drainage efficiency as a process readout linked to A*β* load and the pathological endpoints of CAA. Other conditions, including Parkinson′s disease, autism spectrum disorder, and depressive disorders, are discussed only briefly because their dominant gut–brain mechanisms may differ and are not consistently captured by the same minimal readout set; conditions such as Parkinson′s disease, autism spectrum disorder, and depressive disorders were only briefly discussed. These requirements and possible falsifiable paths are explained in detail (Table S3).

## 6. Translational Implications: Stratified Evaluation, Human Evidence, and Safety

The gut microbiome is a crucial entry point for changes within the gut–immune–brain axis because it can be rapidly affected by diet, medication, and lifestyle [89]. However, it becomes less practical to summarize efficacy using a single outcome as the number of interventions increases. Rather, the three main issues of whether the intervention activates the intended mechanism (mechanism readout), who is most likely to benefit (stratification), and whether this change results in clinically relevant endpoints (endpoint alignment) should be used to assess effectiveness [90]. Using the framework of “minimum indicator combination–priority intervention–safety monitoring,” this chapter prioritizes mechanism readout over clinical endpoints to assess the strategy′s viability early on. To counteract the prevalent bias of “biomarker improvement but clinical inactivity,” the assessment was broken down into three sequential phases. Within eight–12 weeks, the first step is to determine whether the mechanism readout is in the desired direction. Second, we determine whether humoral or imaging signs moved in the same direction as the mechanism of action. Third, the functional scales and clinical outcomes should be confirmed. Instead of discrediting the intervention plan itself, a mechanism readout that does not match the forecasts typically suggests a stratification mismatch or improper dose/timing. Instead of rejecting the suggested mechanism chain, if the mechanism readout is positive but the clinical endpoints lag, it indicates the need for longer follow‐up, more sensitive functional endpoints, or better‐aligned outcome levels (Table 2). Importantly, the translational readiness of these strategies remains uneven. Many mechanisms discussed above are supported primarily by animal, germ‐free, antibiotic‐treated, genetic, or in vitro models, whereas human evidence is often modest, heterogeneous, or based on surrogate endpoints. Therefore, the interventions discussed below should not be interpreted as clinically established applications of the gating failure framework. Instead, they are organized according to the current level of human support, target engagement, reproducibility, safety, and endpoint alignment.

**Table 2:** Stratification–intervention–endpoint alignment: minimal indicator sets for gating failure.

Stratified subtype (dominant gating layer)	Minimal trackable indicator set	Intervention priority (what to do first)	Endpoint alignment (mechanism first, then clinical)
A. Upstream input shift/peripheral priming (gut barrier + immune set‐point)	Plasma LBP/sCD14 + hsCRP/IL‐6 + I‐FABP or fecal calprotectin [91, 92]	First, repair the barrier and reduce endotoxemia: high‐fiber diet/prebiotics; then, postbiotics or defined consortia; address concomitant enteritis/metabolic load first when present	8–12 weeks: LBP/sCD14 declines in parallel with CRP. Midterm: neuroinflammation imaging/fluid readouts decrease; clinical endpoints depend on cohort
B. Active boundary adaptive immunity (meningeal immune niche/TLS; prioritize MS)	CSF CXCL13/IgG index or OCB + sNfL + MRI: new Gd‐enhancing lesions or LMCE (leptomeningeal contrast enhancement) [93, 94]	First suppress meningeal adaptive immunity and interrupt TLS: standard immunotherapy as baseline; meanwhile, correct gut‐driven Th17/Tfh skewing (diet/metabolism/targeted microbiome strategies)	8–12 weeks: CXCL13/sNfL decreases or stabilizes; fewer enhancing lesions. Midterm: reduced relapse rate/lesion burden; improved clinical scales
C. Low‐efficiency clearance gating/protein‐load sensitive (mLVs/glymphatic; prioritize AD)	CSF A*β*42/40, p‐tau + MRI: PVS/DTI‐ALPS (diffusion tensor image analysis along the perivascular space; glymphatic proxy) or tracer‐based clearance + plasma/CSF NfL [95, 96]	First restore clearance and vascular–boundary homeostasis: sleep/circadian optimization + vascular risk management; lifestyle and metabolic support to promote CSF–lymph drainage; add anti‐A*β*/antitau when indicated	8–12 weeks: improved clearance imaging/fluid kinetics; A*β*/p‐tau trends stabilize. Mid‐term: slower protein‐burden progression; delayed cognitive/functional endpoints

### 6.1. Live Bacteria Intervention: Assessing Efficacy and Safety from Psychobiotics to Next‐Generation Probiotics

Live bacterial formulations that have been found to affect mood, stress response, sleep, or cognitive indications are referred to as “psychobiotics” [95]. Importantly, these effects are highly strain‐specific and depend on the host background. Although animal studies have shed light on possible pathways, there is still a lack of reliable human applications of these discoveries [96]. For example, alterations in central receptor expression and anxiety‐ and depression‐like behaviors in rats have been associated with Lactobacillus rhamnosus JB‐1, indicating the involvement of the vagus nerve system. Nevertheless, rather than reproducible clinical results in humans, these findings mainly indicate possible mechanistic routes rather than reproducible clinical efficacy in humans [97]. Thus, psychobiotics should currently be viewed as candidate adjunctive tools with strain‐ and host‐specific effects, rather than as validated microbiota‐based therapies for core neurological disease modification. Randomized, double‐blind controlled trials that identify individual strains are rare at the population study level, and the results usually concentrate on “mild to moderate” markers such as stress, sleep, or quality of life rather than conclusive changes in core illness outcomes. For instance, Bifidobacterium longum 1714 has demonstrated some improvements in brain function, sleep/quality of life, and stress‐related markers; however, consistency across studies and endpoints is lacking [98]. Overall, the available data support the use of live bacterial preparations as a supplemental tool. Although they may modify symptoms and sensitivity to stress, sleep, and mood, they are not sufficient to show appreciable changes in hard endpoints or core disease processes. “Next‐Generation Probiotics (NGPs)” broaden the scope of potential microorganisms beyond conventional lactic acid bacteria and bifidobacteria to include symbiotic bacteria with particular functional goals, including *Faecalibacterium prausnitzii* and *Akkermansia muciniphila*. These NGPs provide more precise target and route orientations and are frequently associated with immune control, metabolic balance, and barrier support. Nonetheless, the standards for safety evaluation and quality control have increased, bringing management closer to “live biotherapeutic products” [99, 100]. For instance, although *Akkermansia* is commonly credited with metabolic advantages, its dependence on mucin as a substrate raises concerns in hosts with compromised mucus layers, barriers, or heightened susceptibility to infection translocation. Therefore, infection risk and barrier status must be considered necessary safety requirements and classification factors [101]. Thus, the existence of a strain function and its ability to be triggered and maintained within a manageable risk threshold under a particular host condition are both necessary for the viability of NGPs.

### 6.2. Evolution of Fecal Microbiota Transplantation (FMT): Towards “Postbiotics” and “Specific Microbiota”

The treatment of recurrent or refractory *Clostridium difficile* infection (rCDI) now has the best evidence and the most obvious therapeutic approach for FMT [102]. Regulatory and consensus guidelines in the US and Europe generally suggest FMT as a therapeutic option after repeated relapses or when normal anti‐infection regimens fail. As essential safety precautions, these protocols stress the significance of donor screening, consistent preparation, sample retention and traceability, and long‐term follow‐up [103]. The necessity of “process controllability and risk minimization” in FMT procedures has been emphasized by ongoing regulatory warnings concerning major adverse events, such as the possibility of spreading drug‐resistant bacteria and diseases. Most FMT research pertaining to the neurological system is still experimental, in contrast to the well‐defined pathway for rCDI. Therefore, robust therapeutic conclusions from rCDI should not be generalized to neuroinflammatory or neurodegenerative disorders without disease‐specific randomized trials and standardized donor, preparation, dosing, and safety procedures. The establishment of reproducible therapeutic pathways is hampered by issues such as cohort heterogeneity, differences in donors and preparation techniques, irregular administration routes and frequencies, and a variety of endpoint selections. Using FMT as a mechanism verification tool under stringent supervision and quality systems to ascertain “which microbial functional signals can be stably triggered and align with measurable readouts under specific host stratification” [104, 105] is a more sensible strategy for transformation. Recently, breaking down the “whole‐stool transfer/input” into a more manageable and producible form has shown greater promise. This includes methods such as further characterizing microbiota complexes and live biological therapeutic products or washing microbiota transplantation (WMT), which improves consistency through standardized isolation and washing procedures. Furthermore, the creation of “postbiotics,” which do not include live bacteria but still contain useful components, attempts to improve repeatability while preserving essential activities and lowering the risk of infection and the spread of drug resistance [106].

### 6.3. Dietary Intervention and Prebiotics: Scalable Upstream Strategies for Microecology

Diet is a major determinant of the substrates and energy sources available to the intestinal microecology and an upstream element that affects the structure and metabolic output of the microbiota. At the population level, it is the most accessible and economical intervention [107]. According to available data, “dietary patterns,” as opposed to specific items, ought to be the main topic of conversation because they guarantee a steady flow of fermentable substrates, stabilize the metabolite profile, and fortify the link with the inflammatory background [108]. Prebiotics and dietary fiber are frequently highlighted. Increased fiber consumption often promotes the preservation of SCFA‐related functional niches and may be associated with a decrease in peripheral inflammatory burden. The amount of fermentable substrates, predictability of the fermentation site‐to‐product ratio, and stability of changes in metabolic output are all affected by the wide variations in fiber types (soluble/insoluble, oligosaccharides of different structures), sources, and doses [109]. Therefore, rather than using a straightforward “supplement or not” choice, fiber/prebiotic therapies should be framed within a “dose‐tolerance‐metabolic readout” model. Fermented meals are also an option. Although dietary baseline, fermentation types, and consumption consistency have a substantial impact on this association, some longitudinal studies have connected them to a decrease in inflammation‐related proteins and an increase in gut microbial diversity. To guarantee consistency and repeatability across cohorts, larger sample sizes and stricter controls are required [110, 111]. Instead of concentrating only on fermentation, the goal of incorporating fermented foods into intervention techniques should be to obtain repeatable functional readouts under controlled consumption conditions. The ketogenic diet represents one of the more clinically established microbiota‐relevant interventions, particularly in epilepsy, although its efficacy cannot be generalized to most neurodegenerative or psychiatric conditions. Although short‐term mechanisms show promise, real‐world applications may falter due to safety or sustainability concerns if nutritional risks, metabolic side effects, and long‐term compliance are not equally evaluated when considering their use for emotional disorders or neurodegenerative conditions [112, 113]. In general, rather than directly changing core neurological outcomes in the short term, dietary interventions are more likely to provide a stable physiological basis for later medication, neuroregulation, or psychological interventions by stabilizing metabolic output and lowering peripheral inflammation and metabolic fluctuations [114–116]. Diet/prebiotics are therefore best suited as upstream background corrections and foundational interventions in the stratified‐readout‐endpoint progression of this chapter: first, confirm target engagement using mechanistic and metabolic readouts, then determine whether imaging/humoral indicators change appropriately, and finally, assess at the clinical endpoint verification level.

### 6.4. VNS and Bioelectronic Medicine: Parameter Selection, Subject Stratification and Endpoint Alignment

It is no longer necessary to initially “reshape the microbiota” because VNS directly modulates the autonomic neuro–immune reflex and the emotion/stress network, providing a “loop‐level” therapeutic pathway [117]. Long‐term follow‐up data support the safety and viability of implantable VNS in refractory depressive disorders, and it has been used for drug‐resistant epilepsy [118]. VNS is important for the gut–immune–brain axis because it offers a controllable input to evaluate the causal relationship between the “peripheral inflammatory set point–boundary immune ecology–central network state” [119], not because it adds another pathway to the system. The main obstacles to its progress are inconsistent effects and challenges associated with cross‐study comparison. This discrepancy has three distinct causes and is not coincidental. First, the stimulus parameters (frequency, pulse width, duty cycle/daily dose, stimulus side, and intensity) do not have a consistent “target engagement” across trials [120]. Second, there are significant differences across participants in terms of autonomic nerve tension, comorbidities, baseline inflammatory phenotypes, and medication use, which may result in different central and immunological responses to the same parameter inputs [121]. Third, because there is no traceable layer between “circuit modulation” and “clinical benefits,” endpoint selection is out of step with the mechanism chain [122]. Thus, the next step for VNS should be to align endpoints, readouts, and stratification, rather than increasing indications. A basic indicator set was used to describe the baseline autonomic nerve and inflammatory burden at enrollment. Repeating the targets during the intervention ensured that the stimulus set off the anticipated reflex pathway. To connect parameter selection and clinical outcomes to a testable and comparable causal chain, immune/boundary structure readouts and central network readouts that correspond to this pathway can be used as mediating endpoints. Although VNS has established clinical uses in selected neurological and psychiatric indications, its role as a gut–immune–brain intervention remains investigational. Future trials should distinguish general neuromodulatory effects from gut‐linked immune or barrier‐related target engagement.

### 6.5. Drug Intervention: Retargeting Metabolic Drugs and Gut Orientation Strategies

**Figure 5:**
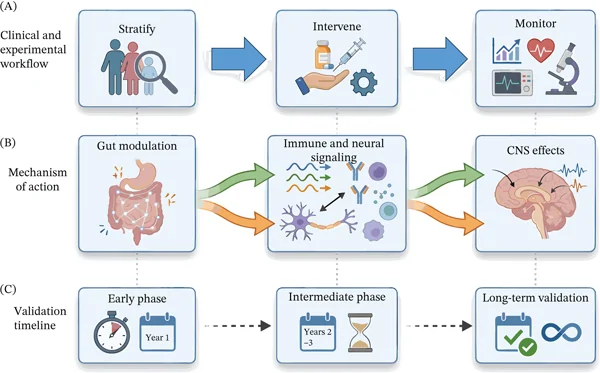
Translational strategy framework for gut–immune–vagal modulation of CNS neuroinflammation. (A) Three‐step workflow: Patient stratification, precision intervention selection, and safety monitoring are performed. (B) Gut‐directed bioelectronic (VNS) and metabolic/pharmacological strategies reshape SCFAs/neuroactive metabolites, strengthen barrier function, and reprogram peripheral macrophage/T‐cell cytokine signaling transmitted via blood/lymphatics and the vagus nerve to influence BBB signaling, microglial activation, neuroinflammation, and neural network plasticity. (C) Staged validation from early mechanism readouts (8–12 weeks) to intermediate imaging/biofluid markers and long‐term clinical endpoints.

Metabolic medications for neurological conditions are increasingly being relocated to other departments. Owing to their widespread clinical use and high accessibility, glucagon‐like peptide‐1 receptor agonists (GLP‐1RAs) have garnered considerable interest in neurodegenerative and cognitive impairment studies [123]. According to recent research, a single pathway may not be responsible for all possible effects. Rather, they most likely stem from a confluence of indirect control of glial cell reactivity, decreased peripheral inflammation, and decreased metabolic load. However, their ability to provide “perceptible” critical endpoint gains for patients within a particular disease subtype and course window will determine their real translational value [124, 125]. Improvements in peripheral indicators may not result in clinical outcomes without such changes. Accordingly, GLP‐1 receptor agonists and other metabolic drugs should be discussed as repurposing candidates requiring disease‐specific clinical validation, not as established gut–brain therapies. Simultaneously, more “targeted” gut‐oriented routes are being investigated, such as detoxification and antimicrobial tactics. According to the gut–immune–brain axis concept, antibiotic intervention should be considered a risk‐benefit strategy for certain circumstances rather than a generic, long‐term intervention, even though it can provide definite advantages in some situations. The pressure of drug resistance selection, long‐term ecological effects, and the possibility of rebound after drug discontinuation are fundamental uncertainties. Furthermore, there are not enough reliable and consistent clinical data to suggest whether the use of antibiotics in combination with prebiotics, probiotics, or postbiotics can reduce risks while maintaining functional advantages [126] (Figure 5).

## 7. Challenges and Future Prospects

Future studies should move from association testing to context‐specific model testing. The key task is to determine when gut‐derived signals, boundary immune changes, clearance impairment, and central glial responses occur in a reproducible order, and when neural, endocrine, vascular, or direct metabolic pathways better explain the findings. The framework should therefore be supported only by temporal ordering, intervention reversibility, anatomical localization, and aligned molecular, imaging, and functional endpoints.

### 7.1. Barrier–Immune Coupling: Neuroinflammation Is a “Gated” Spatial pProcess

The main unresolved question is not whether CNS boundaries participate in gut–brain communication, but which boundary component changes first and under what disease context [127, 128]. Future studies should distinguish increased peripheral input, altered boundary translation, impaired clearance, and glial threshold drift as separable but interacting failure modes [129–133]. These modes correspond, respectively, to intestinal barrier and peripheral immune input, meningeal or BBB/BCSFB signal processing, CSF–lymphatic drainage, and glial‐state remodeling. This distinction is essential because the same microbiota–inflammation association may reflect different mechanisms, including barrier leakage, reduced CSF–lymphatic drainage, vascular dysfunction, or secondary neurodegeneration‐related clearance failure. These failure modes and their minimum testable readouts are summarized in Box 1.

**Box 1:** Testable predictions and minimal readouts for gating failure.

Testable prediction (falsifiable)	Minimal readouts
Prediction 1: Gating failure has a temporal order. If the gut barrier/immune set‐point is an upstream driver, drift in peripheral endotoxin burden and inflammatory set‐point should precede (or at least lead concordantly with) central inflammatory readouts in CSF and imaging. [146, 147]	Plasma LBP/sCD14 + hsCRP/IL‐6, then compare the temporal changes in CSF cytokines/TSPO‐PET or sNfL.
Prediction 2 (AD as the primary battlefield): When clearance gating is the key bottleneck, a decline in clearance kinetics should occur before the accelerated accumulation of the protein burden and should align more closely with vascular endpoints, such as CAA/microbleeds. [134, 148]	MRI PVS/DTI‐ALPS or tracer‐based clearance kinetics + CSF A*β*42/40 and p‐tau; vascular imaging endpoints (microbleeds/CAA) as parallel anchors.
Prediction 3 (MS as primary battlefield): When boundary adaptive immunity is “fixed” into meningeal TLS, CSF B‐cell recruitment signals and meningeal inflammatory imaging should precede or coincide with relapse/new lesions and can serve as stratification and response readouts. [135]	CSF CXCL13/IgG index or OCB + sNfL; MRI new Gd enhancement and leptomeningeal contrast enhancement (LMCE).
Prediction 4: Threshold‐tuning interventions (e.g., SCFA‐related) are more likely to change glial response curves and boundary background before they alter final protein burden/lesion volume. [136]	SCFA levels + glial/boundary background readouts (TSPO‐PET or CSF inflammatory panels and microglial homeostatic gene modules), with structural endpoints assessed in parallel.
Prediction 5: Bone marrow priming can be captured by “near‐field readouts.” If chronic low‐grade gut‐derived endotoxemia persists, coordinated drifts in calvarial bone marrow and meningeal myeloid composition/activation should be traceable and align more with boundary inflammation. [137]	TSPO‐PET or spatial omics in calvarial marrow/meninges; peripheral myeloid bias (monocyte/neutrophil proportions and transcriptional signatures) + CSF/meningeal inflammatory readouts.

### 7.2. Shift in Therapeutic Objectives: From “Anti‐Inflammatory/Clearance” to “Recovery Gating”

Effective treatment, according to the “gated failure” approach, entails more than lowering the protein load or controlling inflammation at a particular location. To restore the regulatory functions of various interfaces, it is necessary to manage peripheral input within a buffering range, re‐establish clearance and isolation activities of border structures, and move glial cells from a locked pathogenic state to a flexible protective path. Three feasible routes should be prioritized when establishing intermediate endpoints. First, intestinal mucosal barrier repair can reset the bone marrow and peripheral immunological set points and decrease the endotoxin‐related input. In addition to lowering inflammatory indicators, this also lessens the proinflammatory bias that reaches the brain border by diminishing the “training effect” of persistent low‐intensity microbial signals on the myeloid system. The reversibility of peripheral myeloid phenotypes (proportion and transcriptional signature), endotoxin load indicators (such as LBP/sCD14), permeability and barrier injury indicators (such as tight junction/epithelial damage markers), and the establishment of a temporal sequence with central boundary inflammation readouts are important transformation endpoints [134, 135]. Second, to restore “drainage‐clearance gating,” the exchange of meningeal lymph and lymphatic substances must be maintained or restarted. Deposition kinetics and border inflammation are affected by drainage efficiency, which controls the timely excretion of inflammatory mediators and misfolded proteins in the brain. Whether improved drainage is associated with a reduced glial response threshold, faster protein clearance, and less border inflammation is a valid hypothesis. The long‐term net benefits of these methods under specific dose windows and safety limits must be evaluated, as they frequently have systemic effects on the vascular and lymphatic system [136–138]. Third, glial state reversal can be achieved through epigenetic modifications. It can be challenging for microglia to spontaneously revert to a stable pathogenic state along the DAM/MGnD trajectory in the presence of proteinopathy and chronic inflammation. In addition to “anti‐inflammation,” strategies that concentrate on chromatin accessibility and transcriptional thresholds (such as HDAC regulatory pathways) also seek to improve state transition plasticity, which permits the rebuilding of phagocytosis, lipid processing, and barrier protective programs [139, 140]. Verifiable endpoints should evaluate whether transitions have taken place, such as whether the phagocytic/lipid metabolism program has resumed, whether the homeostatic module (via gene monitoring) has recovered, and whether the spatial structure surrounding the lesion has changed from a diffuse inflammatory hotspot to a more ordered protective boundary, improving in accordance with neuronal excitability and network plasticity readouts.

### 7.3. Requirements for Replicable Translational Testing

Replicable translational testing requires three elements. First, longitudinal studies should record diet, medication exposure, infection, sleep, and eating patterns to determine whether changes begin at intestinal, peripheral, boundary, or central levels [141, 142]. Second, mechanistic readouts should be cell‐type and state‐specific, supported by single‐cell, spatial, or blocking approaches where possible [143]. Third, cross‐center indicators are needed to identify subgroups in which microbiota‐related or boundary‐related interventions are most likely to be transferable [144]. Peripheral immunophenotypes, barrier‐related readouts, important metabolites, and microbiota function are all focal points of the stratified minimum set. To ensure that “changes in gating status” and “alterations in clinical outcomes” are evaluated within the same framework, boundary structure readouts or physiological endpoints should be included. Integrating cellular state trajectories, chemical mechanisms, and anatomical gatekeeping into a testable causal sequence leads to true advancements rather than general narratives [145]. As part of the common objective of “recovery gating,” barrier repair, boundary drainage function restoration, and glial state reversal are viewed as traceable, intervenable, and verifiable processes. This method increases the likelihood that the effect of the intestine on the brain will be converted from a correlation to reproducible neuropathological evidence.

### 7.4. Critical Evidence Gaps and Competing Interpretations

Several limitations prevent the gating failure framework from being interpreted as an established mechanism. First, much of the strongest causal evidence comes from animal tracing, depletion, antibiotic, germ‐free, or genetic‐manipulation models, whereas human studies often remain cross‐sectional or rely on peripheral proxy markers [28, 141–143]. Second, microbiota changes may be causes, modifiers, or consequences of neurological disease, and are frequently confounded by diet, medication exposure, frailty, sleep disturbance, vascular risk, and disease stage [10, 11, 144]. This is particularly relevant to proposed links between microbial metabolites and glial‐state transitions, TMAO and cerebrovascular pathology, or SCFAs and transcriptional threshold regulation. Third, negative or inconsistent intervention trials indicate that mechanistic plausibility does not guarantee clinical efficacy, as illustrated by variable findings for probiotics, FMT, GLP‐1 receptor agonists, and VNS [98, 105, 122, 124]. Finally, CNS boundary dysfunction may be primary in some contexts but secondary in others, particularly in advanced neurodegeneration or vascular disease [13–16]. Future studies should therefore compare the gating model with competing explanations using longitudinal sampling, disease‐stage stratification, cell‐type‐specific readouts, intervention reversibility, and clinically meaningful endpoints [141–145]. For this reason, translational claims should be graded according to the level of human evidence, distinguishing clinically established contexts such as ketogenic diet in epilepsy from exploratory microbiota‐targeted or barrier‐directed strategies in neurodegenerative and neuroinflammatory diseases. A comparative summary of supportive evidence, limiting findings, and alternative interpretations is provided in Table S4.

## 8. Conclusion

This review proposes barrier–immune coupled gating failure as a working hypothesis for interpreting selected forms of gut–brain neuroinflammation. Rather than serving as a universal explanation, the framework organizes evidence related to CNS boundary regulation, meningeal immunity, skull bone marrow–meningeal communication, BBB/BCSFB function, and CSF–lymphatic clearance. In disease‐specific contexts such as AD and MS, gut‐derived signals are viewed as modulators of clearance, inflammatory thresholds, immune amplification, or disease progression, rather than as primary drivers overriding central proteinopathy, neurodegeneration, genetic susceptibility, or CNS‐specific autoimmunity. The major value of this framework is not to restate that the gut and brain communicate, but to define testable failure modes at CNS interfaces. Future studies should determine whether gut‐related signals temporally precede boundary dysfunction, clearance impairment, or glial‐state remodeling, and whether these changes can be reversed by targeted interventions. Until such evidence is available, barrier–immune gating failure should be treated as a hypothesis‐generating and stratification framework rather than an established therapeutic model.

**Nomenclature**
AD: Alzheimer′s disease
AhR: aryl hydrocarbon receptor
AQP4: aquaporin‐4
BAMs: boundary‐associated macrophages
BBB: blood–brain barrier
BCSFB: blood–cerebrospinal fluid barrier
CAA: cerebral amyloid angiopathy
CNS: central nervous system
CSF: cerebrospinal fluid
DAM: disease‐associated microglia
EAE: experimental autoimmune encephalomyelitis
FMT: fecal microbiota transplantation
GALT: gut‐associated lymphoid tissue
GLP‐1: glucagon‐like peptide‐1
GLP‐1RA: glucagon‐like peptide‐1 receptor agonist
HDAC: histone deacetylase
mLVs: meningeal lymphatic vessels
MS: multiple sclerosis
PVS: Virchow–Robin perivascular space
SCFAs: short‐chain fatty acids
TCVs: transcortical vascular channels
TLS: tertiary lymphoid structures
TMAO: trimethylamine N‐oxide
VNS: vagus nerve stimulation.

## Author Contributions

J.L.: data curation, formal analysis, methodology, resources, software, writing—original draft, and funding acquisition. Y.C.: conceptualization, data curation, investigation, supervision, validation, visualization, and writing—review and editing. F.Q. and Y.L.: methodology, resources, software, validation, visualization, and writing—review and editing. X.X. and Z.Y.: conceptualization, methodology, project administration, and writing—review and editing. W.W.: conceptualization, funding acquisition, project administration, and writing—review and editing. J.L., Y.C., and Y.L. contributed equally to this work.

## Funding

No funding was received for this manuscript.

## Ethics Statement

This article is a review and does not contain any studies with human participants or animals performed by any of the authors. Therefore, ethical approval was not required.

## Consent

The authors have nothing to report.

## Conflicts of Interest

The authors declare no conflicts of interest.

## Supporting Information

Additional supporting information can be found online in the Supporting Information section.

## Supporting information


**Supporting Information 1** Table S1: Mechanistic checkpoints: Evidence tiering, minimal readouts, and confounders (Sections 2–4). It summarizes evidence‐tier boundaries, testable propositions, minimal readouts, major confounders, and translational levers across the barrier, clearance, metabolic, and immune‐cell gating mechanisms addressed in Sections 2–4. Table S2: Disease spectrum and translational strategies: Evidence tiering, minimal readouts, and confounders (Sections 5–6). It summarizes disease‐ and intervention‐oriented applications of the gating framework, including evidence tiers, mechanistic readouts, major confounders, and potential stratification and intervention strategies.

**Supporting Information 2** Table S3 (extended): Additional disease‐specific gating cues and minimal readouts (not expanded in the main text). It provides additional disease‐specific gating layers, key mechanistic cues, and minimal readouts for Parkinson′s disease, autism spectrum disorder, and major depressive disorder, together with the rationale for their limited discussion in the main text.

**Supporting Information 3** Table S4: Comparative strength, limitations, and alternative interpretations of evidence supporting the gating failure framework. It compares supportive evidence, limiting or conflicting evidence, and alternative interpretations across key mechanistic domains, disease contexts, and intervention strategies relevant to the proposed gating failure framework.

**Supporting Information 4** Box S1: Scope and limitations of the barrier–immune gating failure hypothesis. It defines the purpose, scope, applicability, limitations, and evidence requirements of the barrier–immune gating failure hypothesis, and clarifies the conditions under which gut–brain associations may or may not support the proposed framework.

## Data Availability

Data sharing is not applicable to this article as no new data were created or analyzed in this study.

## References

[1] Proulx S. T. and Engelhardt B. , Central Nervous System Zoning: How Brain Barriers Establish Subdivisions for CNS Immune Privilege and Immune Surveillance, Journal of Internal Medicine. (2022) 292, no. 1, 47–67, 10.1111/joim.13469, 35184353. PMC9314672 35184353

[2] Ampie L. and McGavern D. B. , Immunological Defense of CNS Barriers Against Infections, Immunity. (2022) 55, no. 5, 781–799, 10.1016/j.immuni.2022.04.012, 35545028. PMC9087878 35545028

[3] Loh J. S. , Mak W. Q. , Tan L. K. S. , Ng C. X. , Chan H. H. , Yeow S. H. , Foo J. B. , Ong Y. S. , How C. W. , and Khaw K. Y. , Microbiota-Gut-Brain Axis and Its Therapeutic Applications in Neurodegenerative Diseases, Signal Transduction and Targeted Therapy. (2024) 9, no. 1, 10.1038/s41392-024-01743-1, 38360862. PMC10869798 38360862

[4] Chenghan M. , Wanxin L. , Bangcheng Z. , Yao H. , Qinxi L. , Ting Z. , Xiaojie L. , Kun Z. , Yingqian Z. , and Zhihui Z. , Short-Chain Fatty Acids Mediate Gut Microbiota-Brain Communication and Protect the Blood-Brain Barrier Integrity, Annals of the New York Academy of Sciences. (2025) 1545, no. 1, 116–131, 10.1111/nyas.15299, 39998158. 39998158

[5] Li Z. , Zhang F. , Sun M. , Liu J. , Zhao L. , Liu S. , Li S. , and Wang B. , The Modulatory Effects of Gut Microbes and Metabolites on Blood-Brain Barrier Integrity and Brain Function in Sepsis-Associated Encephalopathy, Peer J. (2023) 11, e15122, 10.7717/peerj.15122, 37009158. PMC10064995 37009158

[6] Xu H. , Lotfy P. , Gelb S. , Pragana A. , Hehnly C. , Byer L. I. J. , Shipley F. B. , Zawadzki M. E. , Cui J. , Deng L. , Taylor M. , Webb M. , Lidov H. G. W. , Andermann M. L. , Chiu I. M. , Ordovas-Montanes J. , and Lehtinen M. K. , The Choroid Plexus Synergizes With Immune Cells During Neuroinflammation, Cell. (2024) 187, no. 18, 4946–4963, 10.1016/j.cell.2024.07.002, 39089253. PMC11458255 39089253

[7] Liu L. , Zhang X. , Chai Y. , Zhang J. , Deng Q. , and Chen X. , Skull Bone Marrow and Skull Meninges Channels: Redefining the Landscape of Central Nervous System Immune Surveillance, Cell Death & Disease. (2025) 16, no. 1, 10.1038/s41419-025-07336-2, 39875352. PMC11775313 39875352

[8] Zhang X. , Liu L. , Chai Y. , Zhang J. , Deng Q. , and Chen X. , Reimagining the Meninges From a Neuroimmune Perspective: A Boundary, but Not Peripheral, Journal of Neuroinflammation. (2024) 21, no. 1, 10.1186/s12974-024-03286-2, 39548515. PMC11568633 39548515

[9] Cugurra A. , Mamuladze T. , Rustenhoven J. , Dykstra T. , Beroshvili G. , Greenberg Z. J. , Baker W. , Papadopoulos Z. , Drieu A. , Blackburn S. , Kanamori M. , Brioschi S. , Herz J. , Schuettpelz L. G. , Colonna M. , Smirnov I. , and Kipnis J. , Skull and Vertebral Bone Marrow Are Myeloid Cell Reservoirs for the Meninges and CNS Parenchyma, Science. (2021) 373, no. 6553, eabf7844, 10.1126/science.abf7844, 34083447. PMC8863069 34083447

[10] Bruggeman A. , Vandendriessche C. , Hamerlinck H. , De Looze D. , Tate D. J. , Vuylsteke M. , De Commer L. , Devolder L. , Raes J. , Verhasselt B. , and Laukens D. , Safety and Efficacy of Faecal Microbiota Transplantation in Patients With Mild to Moderate Parkinson′s Disease (GUT-PARFECT): A Double-Blind, Placebo-Controlled, Randomised, Phase 2 Trial, EClinicalMedicine. (2024) 71, 102563, 10.1016/j.eclinm.2024.102563, 38686220. PMC11056595 38686220

[11] Lo S. W. , Hung T. H. , Lin Y. T. , Lee C. S. , Chen C. Y. , Fang C. J. , and Lai P. C. , Clinical Efficacy and Safety of Faecal Microbiota Transplantation in the Treatment of Irritable Bowel Syndrome: A Systematic Review, Meta-Analysis and Trial Sequential Analysis, European Journal of Medical Research. (2024) 29, no. 1, 10.1186/s40001-024-02046-5, 39289768. PMC11409544 39289768

[12] Mokbel A. Y. , Burns M. P. , and Main B. S. , The Contribution of the Meningeal Immune Interface to Neuroinflammation in Traumatic Brain Injury, Journal of Neuroinflammation. (2024) 21, no. 1, 10.1186/s12974-024-03122-7, 38802931. PMC11131220 38802931

[13] Ohara M. and Hattori T. , The Glymphatic System in Cerebrospinal Fluid Dynamics: Clinical Implications, Its Evaluation, and Application to Therapeutics, Neuro-Degenerative Diseases. (2025) 25, no. 4, 189–201, 10.1159/000546286, 40367923. PMC12158400 40367923

[14] Licastro E. , Pignataro G. , Iliff J. J. , Xiang Y. , Lo E. H. , Hayakawa K. , and Esposito E. , Glymphatic and Lymphatic Communication With Systemic Responses During Physiological and Pathological Conditions in the Central Nervous System, Communications Biology. (2024) 7, no. 1, 10.1038/s42003-024-05911-5, 38402351. PMC10894274 38402351

[15] Chen X. , Liu X. , Koundal S. , Elkin R. , Zhu X. , Monte B. , Xu F. , Dai F. , Pedram M. , Lee H. , Kipnis J. , Tannenbaum A. , van Nostrand W. E. , and Benveniste H. , Cerebral Amyloid Angiopathy Is Associated With Glymphatic Transport Reduction and Time-Delayed Solute Drainage Along the Neck Arteries, Nature Aging. (2022) 2, no. 3, 214–223, 10.1038/s43587-022-00181-4, 36199752. PMC9531841 36199752

[16] Ang P. S. , Zhang D. M. , Azizi S. A. , Norton de Matos S. A. , and Brorson J. R. , The Glymphatic System and Cerebral Small Vessel Disease, Journal of Stroke and Cerebrovascular Diseases: The Official Journal of National Stroke Association. (2024) 33, no. 3, 107557, 10.1016/j.jstrokecerebrovasdis.2024.107557, 38198946. PMC11579894 38198946

[17] Voicu V. , Toader C. , ?erban M. , Covache-Busuioc R. A. , and Ciurea A. V. , Systemic Neurodegeneration and Brain Aging: Multi-Omics Disintegration, Proteostatic Collapse, and Network Failure Across the CNS, Biomedicines. (2025) 13, no. 8, 10.3390/biomedicines13082025, 40868276. PMC12383969 40868276

[18] Wang J. , Zhao Q. , Zhang S. , Liu J. , Fan X. , Han B. , Hou Y. , and Ai X. , Microbial Short Chain Fatty Acids: Effective Histone Deacetylase Inhibitors in Immune Regulation (Review), International Journal of Molecular Medicine. (2025) 57, no. 1, 10.3892/ijmm.2025.5687. PMC12634069 41201037

[19] Caetano-Silva M. E. , Rund L. , Hutchinson N. T. , Woods J. A. , Steelman A. J. , and Johnson R. W. , Inhibition of Inflammatory Microglia by Dietary Fiber and Short-Chain Fatty Acids, Scientific Reports. (2023) 13, no. 1, 10.1038/s41598-022-27086-x, 36797287. PMC9935636 36797287

[20] Li Y. , Liu A. , Chen K. , Li L. , Zhang X. , Zou F. , Zhang X. , and Meng X. , Sodium Butyrate Alleviates Lead-Induced Neuroinflammation and Improves Cognitive and Memory Impairment Through the ACSS2/H3K9ac/BDNF Pathway, Environment International. (2024) 184, 108479, 10.1016/j.envint.2024.108479, 38340407. 38340407

[21] Seo S. K. and Kwon B. , Immune Regulation Through Tryptophan Metabolism, Experimental & Molecular Medicine. (2023) 55, no. 7, 1371–1379, 10.1038/s12276-023-01028-7, 37394584. PMC10394086 37394584

[22] Yang Y. , Wang N. , Xu L. , Liu Y. , Huang L. , Gu M. , Wu Y. , Guo W. , and Sun H. , Aryl Hydrocarbon Receptor Dependent Anti-Inflammation and Neuroprotective Effects of Tryptophan Metabolites on Retinal Ischemia/Reperfusion Injury, Cell Death & Disease. (2023) 14, no. 2, 10.1038/s41419-023-05616-3, 36754954. PMC9908897 36754954

[23] Wu X. , Miller J. A. , Lee B. T. K. , Wang Y. , and Ruedl C. , Reducing Microglial Lipid Load Enhances *β* Amyloid Phagocytosis in an Alzheimer′s Disease Mouse Model, Science Advances. (2025) 11, no. 6, eadq6038, 10.1126/sciadv.adq6038, 39908361. PMC11797491 39908361

[24] Schonhoff A. M. , Figge D. A. , Williams G. P. , Jurkuvenaite A. , Gallups N. J. , Childers G. M. , Webster J. M. , Standaert D. G. , Goldman J. E. , and Harms A. S. , Border-Associated Macrophages Mediate the Neuroinflammatory Response in an Alpha-Synuclein Model of Parkinson Disease, Nature Communications. (2023) 14, no. 1, 10.1038/s41467-023-39060-w, 37365181. PMC10293214 37365181

[25] Hutchinson A. N. , Antonsson A. E. , Forsgård R. A. , König J. , Ganda Mall J. P. , and Rode J. , The Effects of Oral Probiotic Intervention on Brain Structure and Function in Human Adults: A Systematic Review, NPJ Biofilms and Microbiomes. (2026) 12, no. 1, 10.1038/s41522-025-00872-x, 41501074. PMC12779965 41501074

[26] Kim J. H. , Choi Y. , Lee S. , and Oh M. S. , Probiotics as Potential Treatments for Neurodegenerative Diseases: A Review of the Evidence From In Vivo to Clinical Trial, Biomolecules & Therapeutics. (2025) 33, no. 1, 54–74, 10.4062/biomolther.2024.215, 39676295. PMC11704393 39676295

[27] Jafari M. , Alipour M. , Zamani S. , Mohtasham Amiri A. , Pourabbas P. , and Hasannejad-Bibalan M. , Probiotics as a Complementary Medicine in Neurologic Disorders, Health Science Reports. (2025) 8, no. 11, e71422, 10.1002/hsr2.71422, 41163934. PMC12559909 41163934

[28] Vatkar A. , Kale S. , Shyam A. , and Srivastava S. , Understanding the Levels of Evidence in Medical Research, Journal of Orthopaedic Case Reports. (2025) 15, no. 5, 6–9, 10.13107/jocr.2025.v15.i05.5534, 40351623. PMC12064251 40351623

[29] Mazzitelli J. A. , Pulous F. E. , Smyth L. C. D. , Kaya Z. , Rustenhoven J. , Moskowitz M. A. , Kipnis J. , and Nahrendorf M. , Skull Bone Marrow Channels as Immune Gateways to the Central Nervous System, Nature Neuroscience. (2023) 26, no. 12, 2052–2062, 10.1038/s41593-023-01487-1, 37996526. PMC10894464 37996526

[30] Goodman G. W. , Devlin P. , West B. E. , and Ritzel R. M. , The Emerging Importance of Skull-Brain Interactions in Traumatic Brain Injury, Frontiers in Immunology. (2024) 15, 1353513, 10.3389/fimmu.2024.1353513, 38680490. PMC11047125 38680490

[31] García-Montero C. , Fraile-Martínez O. , Gómez-Lahoz A. M. , Pekarek L. , Castellanos A. J. , Noguerales-Fraguas F. , Coca S. , Guijarro L. G. , García-Honduvilla N. , Asúnsolo A. , Sanchez-Trujillo L. , Lahera G. , Bujan J. , Monserrat J. , Álvarez-Mon M. , Álvarez-Mon M. A. , and Ortega M. A. , Nutritional Components in Western Diet Versus Mediterranean Diet at the Gut Microbiota-Immune System Interplay. Implications for Health and Disease, Nutrients. (2021) 13, no. 2, 10.3390/nu13020699, 33671569. PMC7927055 33671569

[32] Zhang Q. , Niu Y. , Li Y. , Xia C. , Chen Z. , Chen Y. , and Feng H. , Meningeal Lymphatic Drainage: Novel Insights Into Central Nervous System Disease, Signal Transduction and Targeted Therapy. (2025) 10, no. 1, 10.1038/s41392-025-02177-z, 40320416. PMC12050339 40320416

[33] El Kamouh M. R. , El Kamouh M. R. , Lenck S. , Lehericy S. , Benveniste H. , and Thomas J. L. , Fluid and Waste Clearance in Central Nervous System Health and Diseases, Neuro-Degenerative Diseases. (2025) 25, no. 3, 145–166, 10.1159/000546018, 40334649. PMC12173435 40334649

[34] Yankova G. , Bogomyakova O. , and Tulupov A. , The Glymphatic System and Meningeal Lymphatics of the Brain: New Understanding of Brain Clearance, Reviews in the Neurosciences. (2021) 32, no. 7, 693–705, 10.1515/revneuro-2020-0106, 33618444. 33618444

[35] Tian Y. , Zhao M. , Chen Y. , Yang M. , and Wang Y. , The Underlying Role of the Glymphatic System and Meningeal Lymphatic Vessels in Cerebral Small Vessel Disease, Biomolecules. (2022) 12, no. 6, 10.3390/biom12060748, 35740873. PMC9221030 35740873

[36] Alavian F. and Hajimohammadi A. , Interplay Between Gut Microbiome and Glymphatic System in Cognitive Function and Memory Regulation, Molecular Biology Reports. (2025) 52, no. 1, 10.1007/s11033-025-11129-3, 41099783. 41099783

[37] Che Mohd Nassir C. M. N. , Che Ramli M. D. , Mohamad Ghazali M. , Jaffer U. , Abdul Hamid H. , Mehat M. Z. , and Hein Z. M. , The Microbiota-Gut-Brain Axis: Key Mechanisms Driving Glymphopathy and Cerebral Small Vessel Disease, Life. (2025) 15, no. 1, 10.3390/life15010003. PMC11766513 39859943

[38] Wu H. , Liu B. , Liu W. V. , Wen Z. , Yang W. , Yang H. , Li J. , and Zha Y. , Glymphatic System Dysfunction Correlated With Gut Dysbiosis and Cognitive Impairment in Schizophrenia, Schizophrenia. (2025) 11, no. 1, 10.1038/s41537-025-00661-7, 40796779. PMC12343894 40796779

[39] Li G. , Cao Y. , Tang X. , Huang J. , Cai L. , and Zhou L. , The Meningeal Lymphatic Vessels and the Glymphatic System: Potential Therapeutic Targets in Neurological Disorders, Journal of Cerebral Blood Flow and Metabolism: Official Journal of the International Society of Cerebral Blood Flow and Metabolism. (2022) 42, no. 8, 1364–1382, 10.1177/0271678X221098145, 35484910. PMC9274866 35484910

[40] Gouveia-Freitas K. and Bastos-Leite A. J. , Perivascular Spaces and Brain Waste Clearance Systems: Relevance for Neurodegenerative and Cerebrovascular Pathology, Neuroradiology. (2021) 63, no. 10, 1581–1597, 10.1007/s00234-021-02718-7, 34019111. PMC8460534 34019111

[41] Sun R. and Jiang H. , Border-Associated Macrophages in the Central Nervous System, Journal of Neuroinflammation. (2024) 21, no. 1, 10.1186/s12974-024-03059-x, 38481312. PMC10938757 38481312

[42] Li T. , Zhang J. , Song H. , Zhang R. , Fan F. , Huang Z. , Zeng M. L. , Peng B. W. , and Zhang J. , Border-Associated Macrophages: An Emerging Perspective From Physiological Basis and Multi-Disease Roles to the Mechanism of Vascular Cognitive Impairment and Dementia, Journal of Neuroinflammation. (2025) 22, no. 1, 10.1186/s12974-025-03631-z, 41469705. PMC12754968 41469705

[43] Jing L. , Zhang H. , Xiang Q. , Shen L. , Guo X. , Zhai C. , and Hu H. , Targeting Trimethylamine N-Oxide: A New Therapeutic Strategy for Alleviating Atherosclerosis, Frontiers in Cardiovascular Medicine. (2022) 9, 864600, 10.3389/fcvm.2022.864600, 35770223. PMC9235870 35770223

[44] Wang Z. , Li X. , Moura A. K. , Hu J. Z. , Wang Y. T. , and Zhang Y. , Lysosome Functions in Atherosclerosis: A Potential Therapeutic Target, Cells. (2025) 14, no. 3, 10.3390/cells14030183. PMC11816498 39936975

[45] Hawkes C. A. and McLaurin J. , Selective Targeting of Perivascular Macrophages for Clearance of Beta-Amyloid in Cerebral Amyloid Angiopathy, Proceedings of the National Academy of Sciences of the United States of America. (2009) 106, no. 4, 1261–1266, 10.1073/pnas.0805453106, 19164591. PMC2633563 19164591

[46] Uekawa K. , Hattori Y. , Ahn S. J. , Seo J. , Casey N. , Anfray A. , Zhou P. , Luo W. , Anrather J. , Park L. , and Iadecola C. , Border-Associated Macrophages Promote Cerebral Amyloid Angiopathy and Cognitive Impairment Through Vascular Oxidative Stress, Molecular Neurodegeneration. (2023) 18, no. 1, 10.1186/s13024-023-00660-1, 37789345. PMC10548599 37789345

[47] Bonnar O. , Eyre B. , and van Veluw S. J. , Perivascular Brain Clearance as a Therapeutic Target in Cerebral Amyloid Angiopathy and Alzheimer′s Disease, Neurotherapeutics : The Journal of the American Society for Experimental Neuro Therapeutics. (2025) 22, no. 3, e00535, 10.1016/j.neurot.2025.e00535, 39890534. PMC12047398 39890534

[48] Mann E. R. , Lam Y. K. , and Uhlig H. H. , Short-Chain Fatty Acids: Linking Diet, the Microbiome and Immunity, Nature Reviews Immunology. (2024) 24, no. 8, 577–595, 10.1038/s41577-024-01014-8. 38565643

[49] Patnala R. , Arumugam T. V. , Gupta N. , and Dheen S. T. , HDAC Inhibitor Sodium Butyrate-Mediated Epigenetic Regulation Enhances Neuroprotective Function of Microglia During Ischemic Stroke, Molecular Neurobiology. (2017) 54, no. 8, 6391–6411, 10.1007/s12035-016-0149-z, 27722928. 27722928

[50] Chang P. V. , Hao L. , Offermanns S. , and Medzhitov R. , The Microbial Metabolite Butyrate Regulates Intestinal Macrophage Function via Histone Deacetylase Inhibition, Proceedings of the National Academy of Sciences of the United States of America. (2014) 111, no. 6, 2247–2252, 10.1073/pnas.1322269111, 24390544. PMC3926023 24390544

[51] Lan Z. , Tang X. , Lu M. , Hu Z. , and Tang Z. , The Role of Short-Chain Fatty Acids in Central Nervous System Diseases: A Bibliometric and Visualized Analysis With Future Directions, Heliyon. (2024) 10, no. 4, e26377, 10.1016/j.heliyon.2024.e26377, 38434086. PMC10906301 38434086

[52] Erny D. , Hrabě de Angelis A. L. , Jaitin D. , Wieghofer P. , Staszewski O. , David E. , Keren-Shaul H. , Mahlakoiv T. , Jakobshagen K. , Buch T. , Schwierzeck V. , Utermöhlen O. , Chun E. , Garrett W. S. , McCoy K. D. , Diefenbach A. , Staeheli P. , Stecher B. , Amit I. , and Prinz M. , Host Microbiota Constantly Control Maturation and Function of Microglia in the CNS, Nature Neuroscience. (2015) 18, no. 7, 965–977, 10.1038/nn.4030, 26030851. PMC5528863 26030851

[53] Cook J. and Prinz M. , Regulation of Microglial Physiology by the Microbiota, Gut Microbes. (2022) 14, no. 1, 2125739, 10.1080/19490976.2022.2125739, 36151874. PMC9519021 36151874

[54] Sorgdrager F. J. H. , Naudé P. J. W. , Kema I. P. , Nollen E. A. , and Deyn P. P. , Tryptophan Metabolism in Inflammaging: From Biomarker to Therapeutic Target, Frontiers in Immunology. (2019) 10, 10.3389/fimmu.2019.02565, 31736978. PMC6833926 31736978

[55] Vázquez-Gómez G. , Karasová M. , Tylichová Z. , Kabátková M. , Hampl A. , Matthews J. , Neča J. , Ciganek M. , Machala M. , and Vondráček J. , Aryl Hydrocarbon Receptor (AhR) Limits the Inflammatory Responses in Human Lung Adenocarcinoma A549 Cells via Interference With NF-*κ*B Signaling, Cells. (2022) 11, no. 4, 10.3390/cells11040707, 35203356. PMC8870046 35203356

[56] Øvrevik J. , Låg M. , Lecureur V. , Gilot D. , Lagadic-Gossmann D. , Refsnes M. , Schwarze P. E. , Skuland T. , Becher R. , and Holme J. A. , AhR and Arnt Differentially Regulate NF-*κ*B Signaling and Chemokine Responses in Human Bronchial Epithelial Cells, Cell Communication and Signaling: CCS. (2014) 12, no. 1, 10.1186/s12964-014-0048-8, 25201625. PMC4222560 25201625

[57] Rothhammer V. , Mascanfroni I. D. , Bunse L. , Takenaka M. C. , Kenison J. E. , Mayo L. , Chao C. C. , Patel B. , Yan R. , Blain M. , Alvarez J. I. , Kébir H. , Anandasabapathy N. , Izquierdo G. , Jung S. , Obholzer N. , Pochet N. , Clish C. B. , Prinz M. , Prat A. , Antel J. , and Quintana F. J. , Type I Interferons and Microbial Metabolites of Tryptophan Modulate Astrocyte Activity and Central Nervous System Inflammation via the Aryl Hydrocarbon Receptor, Nature Medicine. (2016) 22, no. 6, 586–597, 10.1038/nm.4106, 27158906. PMC4899206 27158906

[58] Kaye J. , Piryatinsky V. , Birnberg T. , Hingaly T. , Raymond E. , Kashi R. , Amit-Romach E. , Caballero I. S. , Towfic F. , Ator M. A. , Rubinstein E. , Laifenfeld D. , Orbach A. , Shinar D. , Marantz Y. , Grossman I. , Knappertz V. , Hayden M. R. , and Laufer R. , Laquinimod Arrests Experimental Autoimmune Encephalomyelitis by Activating the Aryl Hydrocarbon Receptor, Proceedings of the National Academy of Sciences of the United States of America. (2016) 113, no. 41, E6145–E6152, 10.1073/pnas.1607843113, 27671624. PMC5068259 27671624

[59] Rothhammer V. , Kenison J. E. , Li Z. , Tjon E. , Takenaka M. C. , Chao C. C. , Alves de Lima K. , Borucki D. M. , Kaye J. , and Quintana F. J. , Aryl Hydrocarbon Receptor Activation in Astrocytes by Laquinimod Ameliorates Autoimmune Inflammation in the CNS, Neurology: Neuroimmunology & Neuroinflammation. (2021) 8, no. 2, 10.1212/NXI.0000000000000946. PMC7862099 33408169

[60] van den Hoogen W. J. , Laman J. D. , and ’t Hart B. A. , Modulation of Multiple Sclerosis and Its Animal Model Experimental Autoimmune Encephalomyelitis by Food and Gut Microbiota, Frontiers in Immunology. (2017) 8, 10.3389/fimmu.2017.01081, 28928747. PMC5591889 28928747

[61] Butcher E. L. and Arthur S. , Emerging Roles of Bile Acids in Neuroinflammation, International Journal of Molecular Sciences. (2025) 26, no. 23, 11301, 10.3390/ijms262311301. PMC12692218 41373461

[62] Zhang M. Y. , Zhu L. , Zheng X. , Xie T. H. , Wang W. , Zou J. , Li Y. , Li H. Y. , Cai J. , Gu S. , Yao Y. , and Wei T. T. , TGR5 Activation Ameliorates Mitochondrial Homeostasis via Regulating the PKC*δ*/Drp 1-HK2 Signaling in Diabetic Retinopathy, Frontiers in Cell and Developmental Biology. (2022) 9, 759421, 10.3389/fcell.2021.759421. PMC8795816 35096809

[63] Romero-Ramírez L. and Mey J. , Emerging Roles of Bile Acids and TGR5 in the Central Nervous System: Molecular Functions and Therapeutic Implications, International Journal of Molecular Sciences. (2024) 25, no. 17, 10.3390/ijms25179279. PMC11395147 39273226

[64] Xing C. , Huang X. , Wang D. , Yu D. , Hou S. , Cui H. , and Song L. , Roles of Bile Acids Signaling in Neuromodulation Under Physiological and Pathological Conditions, Cell & Bioscience. (2023) 13, no. 1, 10.1186/s13578-023-01053-z, 37308953. PMC10258966 37308953

[65] Grundeken E. and El Aidy S. , Enteroendocrine Cells: The Gatekeepers of Microbiome-Gut-Brain Communication, NPJ Biofilms and Microbiomes. (2025) 11, no. 1, 10.1038/s41522-025-00810-x, 40890205. PMC12402511 40890205

[66] Brierley D. I. and de Lartigue G. , Reappraising the Role of the Vagus Nerve in GLP-1-Mediated Regulation of Eating, British Journal of Pharmacology. (2022) 179, no. 4, 584–599, 10.1111/bph.15603, 34185884. PMC8714868 34185884

[67] Barton J. R. , Londregan A. K. , Alexander T. D. , Entezari A. A. , Covarrubias M. , and Waldman S. A. , Enteroendocrine Cell Regulation of the Gut-Brain Axis, Frontiers in Neuroscience. (2023) 17, 1272955, 10.3389/fnins.2023.1272955, 38027512. PMC10662325 38027512

[68] Wong C. K. , McLean B. A. , Baggio L. L. , Koehler J. A. , Hammoud R. , Rittig N. , Yabut J. M. , Seeley R. J. , Brown T. J. , and Drucker D. J. , Central Glucagon-Like Peptide 1 Receptor Activation Inhibits Toll-Like Receptor Agonist-Induced Inflammation, Cell Metabolism. (2024) 36, no. 1, 130–143, 10.1016/j.cmet.2023.11.009, 38113888. 38113888

[69] Yao Z. , van Velthoven C. T. J. , Kunst M. , Zhang M. , McMillen D. , Lee C. , Jung W. , Goldy J. , Abdelhak A. , Aitken M. , Baker K. , Baker P. , Barkan E. , Bertagnolli D. , Bhandiwad A. , Bielstein C. , Bishwakarma P. , Campos J. , Carey D. , Casper T. , Chakka A. B. , Chakrabarty R. , Chavan S. , Chen M. , Clark M. , Close J. , Crichton K. , Daniel S. , DiValentin P. , Dolbeare T. , Ellingwood L. , Fiabane E. , Fliss T. , Gee J. , Gerstenberger J. , Glandon A. , Gloe J. , Gould J. , Gray J. , Guilford N. , Guzman J. , Hirschstein D. , Ho W. , Hooper M. , Huang M. , Hupp M. , Jin K. , Kroll M. , Lathia K. , Leon A. , Li S. , Long B. , Madigan Z. , Malloy J. , Malone J. , Maltzer Z. , Martin N. , McCue R. , McGinty R. , Mei N. , Melchor J. , Meyerdierks E. , Mollenkopf T. , Moonsman S. , Nguyen T. N. , Otto S. , Pham T. , Rimorin C. , Ruiz A. , Sanchez R. , Sawyer L. , Shapovalova N. , Shepard N. , Slaughterbeck C. , Sulc J. , Tieu M. , Torkelson A. , Tung H. , Valera Cuevas N. , Vance S. , Wadhwani K. , Ward K. , Levi B. , Farrell C. , Young R. , Staats B. , Wang M. Q. M. , Thompson C. L. , Mufti S. , Pagan C. M. , Kruse L. , Dee N. , Sunkin S. M. , Esposito L. , Hawrylycz M. J. , Waters J. , Ng L. , Smith K. , Tasic B. , Zhuang X. , and Zeng H. , A high-Resolution Transcriptomic and Spatial Atlas of Cell Types in the Whole Mouse Brain, Nature. (2023) 624, no. 7991, 317–332, 10.1038/s41586-023-06812-z, 38092916. PMC10719114 38092916

[70] Cheng Y. H. and Ho M. S. , Disease-Associated Microglia in Neurodegenerative Diseases: Friend or Foe?, PLoS Biology. (2025) 23, no. 10, e3003426, 10.1371/journal.pbio.3003426, 41118343. PMC12539720 41118343

[71] Wang H. , Microglia Heterogeneity in Alzheimer′s Disease: Insights From Single-Cell Technologies, Frontiers in Synaptic Neuroscience. (2021) 13, 773590, 10.3389/fnsyn.2021.773590, 35002670. PMC8735255 35002670

[72] McFarland K. N. and Chakrabarty P. , Microglia in Alzheimer′s Disease: A Key Player in the Transition Between Homeostasis and Pathogenesis, Neurotherapeutics: The Journal of the American Society for Experimental Neuro Therapeutics. (2022) 19, no. 1, 186–208, 10.1007/s13311-021-01179-3, 35286658. PMC9130399 35286658

[73] Yen J. J. and Yu I. I. , The role of ApoE-mediated Microglial Lipid Metabolism in Brain Aging and Disease, Immunometabolism. (2023) 5, no. 1, e00018, 10.1097/IN9.0000000000000018, 36710921. PMC9869962 36710921

[74] Ribeiro M. , Brigas H. C. , Temido-Ferreira M. , Pousinha P. A. , Regen T. , Santa C. , Coelho J. E. , Marques-Morgado I. , Valente C. A. , Omenetti S. , Stockinger B. , Waisman A. , Manadas B. , Lopes L. V. , Silva-Santos B. , and Ribot J. C. , Meningeal *γδ* T Cell-Derived IL-17 Controls Synaptic Plasticity and Short-Term Memory, Science Immunology. (2019) 4, no. 40, 10.1126/sciimmunol.aay5199, 31604844. PMC6894940 31604844

[75] Wu Y. , Zhang Y. , Yu Y. , Wang X. , Zhen Z. , Yuan Y. , Xie B. , Han M. , Wang M. , Zhang X. , Sun X. , Wen X. , Hashimoto K. , Shang Y. , Yuan S. , and Zhang J. , Small Intestinal *γδ* T17 Cells Promote SAE Through STING/C1q-Induced Microglial Synaptic Pruning in Male Mice, Nature Communications. (2025) 16, no. 1, 10.1038/s41467-025-62181-3, 40702081. PMC12287374 40702081

[76] Feng Y. , He X. , Luo S. , Chen X. , Long S. , Liang F. , Shi T. , Pei Z. , and Li Z. , Chronic Colitis Induces Meninges Traffic of Gut-Derived T Cells, Unbalances M1 and M2 Microglia/Macrophage and Increases Ischemic Brain Injury in Mice, Brain Research. (2019) 1707, 8–17, 10.1016/j.brainres.2018.11.019, 30445026. 30445026

[77] Di Filippo M. , Mancini A. , Bellingacci L. , Gaetani L. , Mazzocchetti P. , Zelante T. , La Barbera L. , De Luca A. , Tantucci M. , Tozzi A. , and Durante V. , Interleukin-17 Affects Synaptic Plasticity and Cognition in an Experimental Model of Multiple Sclerosis, Cell Reports. (2021) 37, no. 10, 110094, 10.1016/j.celrep.2021.110094, 34879272. 34879272

[78] Zipp F. , Bittner S. , and Schafer D. P. , Cytokines as Emerging Regulators of Central Nervous System Synapses, Immunity. (2023) 56, no. 5, 914–925, 10.1016/j.immuni.2023.04.011, 37163992. PMC10233069 37163992

[79] Cheng Y. , Tian D. Y. , and Wang Y. J. , Peripheral Clearance of Brain-Derived A*β* in Alzheimer′s Disease: Pathophysiology and Therapeutic Perspectives, Translational Neurodegeneration. (2020) 9, no. 1, 10.1186/s40035-020-00195-1, 32381118. PMC7204069 32381118

[80] Ding J. , Zhao C. , Hao X. , and Jiao H. , Glymphatic and Meningeal Lymphatic Dysfunction in Alzheimer′s Disease: Mechanisms and Therapeutic Perspectives, Alzheimer′s & Dementia: The Journal of the Alzheimer′s Association. (2025) 21, no. 10, e70709, 10.1002/alz.70709, 41152198. PMC12568399 41152198

[81] Bairamian D. , Sha S. , Rolhion N. , Sokol H. , Dorothée G. , Lemere C. A. , and Krantic S. , Microbiota in Neuroinflammation and Synaptic Dysfunction: A Focus on Alzheimer′s Disease, Molecular Neurodegeneration. (2022) 17, no. 1, 10.1186/s13024-022-00522-2, 35248147. PMC8898063 35248147

[82] Casali B. T. , MacPherson K. P. , Reed-Geaghan E. G. , and Landreth G. E. , Microglia Depletion Rapidly and Reversibly Alters Amyloid Pathology by Modification of Plaque Compaction and Morphologies, Neurobiology of Disease. (2020) 142, 104956, 10.1016/j.nbd.2020.104956, 32479996. PMC7526856 32479996

[83] Luo Y. X. , Yang L. L. , and Yao X. Q. , Gut Microbiota-Host Lipid Crosstalk in Alzheimer′s Disease: Implications for Disease Progression and Therapeutics, Molecular Neurodegeneration. (2024) 19, no. 1, 10.1186/s13024-024-00720-0, 38627829. PMC11020986 38627829

[84] Seo D. O. and Holtzman D. M. , Current Understanding of the Alzheimer′s Disease-Associated Microbiome and Therapeutic Strategies, Experimental & Molecular Medicine. (2024) 56, no. 1, 86–94, 10.1038/s12276-023-01146-2, 38172602. PMC10834451 38172602

[85] Yadav S. K. , Ito K. , and Dhib-Jalbut S. , Interaction of the Gut Microbiome and Immunity in Multiple Sclerosis: Impact of Diet and Immune Therapy, International Journal of Molecular Sciences. (2023) 24, no. 19, 14756, 10.3390/ijms241914756, 37834203. PMC10572709 37834203

[86] Rebeaud J. , Vigne S. , Bressoud V. , Carre R. , Ruiz F. , Peter B. , Phillips N. E. , Siewert L. , Roloff T. , Egli A. , Kuhle J. , Collet T. H. , Pröbstel A. K. , and Pot C. , Gut-Derived Metabolites Drive Th17 Cell Pathogenicity in Multiple Sclerosis, Cell Reports. (2025) 44, no. 10, 116326, 10.1016/j.celrep.2025.116326, 40975867. 40975867

[87] Pikor N. B. , Prat A. , Bar-Or A. , and Gommerman J. L. , Meningeal Tertiary Lymphoid Tissues and Multiple Sclerosis: A Gathering Place for Diverse Types of Immune Cells During CNS Autoimmunity, Frontiers in Immunology. (2016) 6, 10.3389/fimmu.2015.00657. PMC4710700 26793195

[88] Ma X. , Zhang J. , Jiang Q. , Li Y. X. , and Yang G. , Human Microbiome-Derived Peptide Affects the Development of Experimental Autoimmune Encephalomyelitis via Molecular Mimicry, EBioMedicine. (2025) 111, 105516, 10.1016/j.ebiom.2024.105516, 39724786. PMC11732510 39724786

[89] Kee R. , Naughton M. , McDonnell G. V. , Howell O. W. , and Fitzgerald D. C. , A Review of Compartmentalised Inflammation and Tertiary Lymphoid Structures in the Pathophysiology of Multiple Sclerosis, Biomedicines. (2022) 10, no. 10, 10.3390/biomedicines10102604, 36289863. PMC9599335 36289863

[90] O′Riordan K. J. , Moloney G. M. , Keane L. , Clarke G. , and Cryan J. F. , The Gut Microbiota-Immune-Brain Axis: Therapeutic Implications, Cell Reports Medicine. (2025) 6, no. 3, 101982, 10.1016/j.xcrm.2025.101982, 40054458. PMC11970326 40054458

[91] Madison A. A. , Andridge R. , Padin A. C. , Wilson S. , Bailey M. T. , Alfano C. M. , Povoski S. P. , Lipari A. M. , Agnese D. M. , Carson W. E. , Malarkey W. B. , and Kiecolt-Glaser J. K. , Endotoxemia Coupled With Heightened Inflammation Predicts Future Depressive Symptoms, Psychoneuroendocrinology. (2020) 122, 104864, 10.1016/j.psyneuen.2020.104864, 33166799. PMC7721058 33166799

[92] Khademi M. , Kockum I. , Andersson M. L. , Iacobaeus E. , Brundin L. , Sellebjerg F. , Hillert J. , Piehl F. , and Olsson T. , Cerebrospinal Fluid CXCL13 in Multiple Sclerosis: A Suggestive Prognostic Marker for the Disease Course, Multiple Sclerosis Journal. (2011) 17, no. 3, 335–343, 10.1177/1352458510389102, 21135023. 21135023

[93] Harrison D. M. , Allette Y. M. , Zeng Y. , Cohen A. , Dahal S. , Choi S. , Zhuo J. , and Hua J. , Meningeal Contrast Enhancement in Multiple Sclerosis: Assessment of Field Strength, Acquisition Delay, and Clinical Relevance, PloS One. (2024) 19, no. 5, e0300298, 10.1371/journal.pone.0300298, 38809920. PMC11135724 38809920

[94] Jack C. R. , Bennett D. A. , Blennow K. , Carrillo M. C. , Dunn B. , Haeberlein S. B. , Holtzman D. M. , Jagust W. , Jessen F. , Karlawish J. , Liu E. , Molinuevo J. L. , Montine T. , Phelps C. , Rankin K. P. , Rowe C. C. , Scheltens P. , Siemers E. , Snyder H. M. , Sperling R. , Contributors , Elliott C. , Masliah E. , Ryan L. , and Silverberg N. , NIA-AA Research Framework: Toward a Biological Definition of Alzheimer′s Disease, Alzheimer′s & Dementia: The Journal of the Alzheimer′s Association. (2018) 14, no. 4, 535–562, 10.1016/j.jalz.2018.02.018, 29653606. PMC5958625 29653606

[95] Taoka T. , Ito R. , Nakamichi R. , Nakane T. , Kawai H. , and Naganawa S. , Diffusion Tensor Image Analysis Along the Perivascular Space (DTI-ALPS): Revisiting the Meaning and Significance of the Method, Magnetic Resonance in Medical Sciences: MRMS: An Official Journal of Japan Society of Magnetic Resonance in Medicine. (2024) 23, no. 3, 268–290, 10.2463/mrms.rev.2023-0175, 38569866. PMC11234944 38569866

[96] Slykerman R. F. , Davies N. , Vlckova K. , O′Riordan K. J. , Bassett S. A. , Dekker J. , Schellekens H. , Hyland N. P. , Clarke G. , and Patterson E. , Precision Psychobiotics for Gut-Brain Axis Health: Advancing the Discovery Pipelines to Deliver Mechanistic Pathways and Proven Health Efficacy, Microbial Biotechnology. (2025) 18, no. 1, e70079, 10.1111/1751-7915.70079, 39815671. PMC11735468 39815671

[97] Zainal Abidin Z. , Hein Z. M. , Che Mohd Nassir C. M. N. , Shari N. , and Che Ramli M. D. , Pharmacological Modulation of the Gut-Brain Axis: Psychobiotics in Focus for Depression Therapy, Frontiers in Pharmacology. (2025) 16, 1665419, 10.3389/fphar.2025.1665419, 41079735. PMC12511018 41079735

[98] Kelly J. R. , Allen A. P. , Temko A. , Hutch W. , Kennedy P. J. , Farid N. , Murphy E. , Boylan G. , Bienenstock J. , Cryan J. F. , Clarke G. , and Dinan T. G. , Lost in Translation? The Potential Psychobiotic Lactobacillus rhamnosus (JB-1) Fails to Modulate Stress or Cognitive Performance in Healthy Male Subjects, Brain, Behavior, and Immunity. (2017) 61, 50–59, 10.1016/j.bbi.2016.11.018. 27865949

[99] Patterson E. , Tan H. T. T. , Groeger D. , Andrews M. , Buckley M. , Murphy E. F. , and Groeger J. A. , *Bifidobacterium longum* 1714 Improves Sleep Quality and Aspects of Well-Being in Healthy Adults: A Randomized, Double-Blind, Placebo-Controlled Clinical Trial, Scientific Reports. (2024) 14, no. 1, 10.1038/s41598-024-53810-w, 38355674. PMC10866977 38355674

[100] Al-Fakhrany O. M. and Elekhnawy E. , Next-generation probiotics: The Upcoming Biotherapeutics, Molecular Biology Reports. (2024) 51, no. 1, 10.1007/s11033-024-09398-5, 38619680. PMC11018693 38619680

[101] Ahmadi Badi S. , Tavakoli Aval H. , Moradi H. R. , Malek A. , Seyedi S. A. , Davari M. , Bereimipour A. , Khani S. , Khatami S. , and Siadat S. D. , Comparative Study of Liver Injury Protection by *Akkermansia muciniphila* and *Faecalibacterium prausnitzii* Interventions in Live and Cell-Free Supernatant Forms via Targeting the Hepcidin–Ferroportin Axis in Mice With CCl4-Induced Liver Fibrosis, Gut Pathogens. (2025) 17, no. 1, 10.1186/s13099-025-00728-x, 40676686. PMC12273000 40676686

[102] Tingler A. M. and Engevik M. A. , Breaking Down Barriers: Is Intestinal Mucus Degradation by *Akkermansia muciniphila* Beneficial or Harmful?, Infection and Immunity. (2025) 93, no. 9, e0050324, 10.1128/iai.00503-24, 40788133. PMC12418764 40788133

[103] Peery A. F. , Kelly C. R. , Kao D. , Vaughn B. P. , Lebwohl B. , Singh S. , Imdad A. , Altayar O. , and AGA Clinical Guidelines Committee , AGA Clinical Practice Guideline on Fecal Microbiota-Based Therapies for Select Gastrointestinal Diseases, Gastroenterology. (2024) 166, no. 3, 409–434, Electronic address: clinicalpractice@gastro.org 10.1053/j.gastro.2024.01.008, 38395525. 38395525

[104] Keller J. J. , Ooijevaar R. E. , Hvas C. L. , Terveer E. M. , Lieberknecht S. C. , Högenauer C. , Arkkila P. , Sokol H. , Gridnyev O. , Mégraud F. , Kump P. K. , Nakov R. , Goldenberg S. D. , Satokari R. , Tkatch S. , Sanguinetti M. , Cammarota G. , Dorofeev A. , Gubska O. , Ianiro G. , Mattila E. , Arasaradnam R. P. , Sarin S. K. , Sood A. , Putignani L. , Alric L. , Baunwall S. M. D. , Kupcinskas J. , Link A. , Goorhuis A. G. , Verspaget H. W. , Ponsioen C. , Hold G. L. , Tilg H. , Kassam Z. , Kuijper E. J. , Gasbarrini A. , Mulder C. J. J. , Williams H. R. T. , and Vehreschild M. J. G. T. , A Standardised Model for Stool Banking for Faecal Microbiota Transplantation: A Consensus Report From a Multidisciplinary UEG Working Group, United European Gastroenterology Journal. (2021) 9, no. 2, 229–247, 10.1177/2050640620967898, 33151137. PMC8259288 33151137

[105] Vendrik K. E. W. , Ooijevaar R. E. , de Jong P. R. C. , Laman J. D. , van Oosten B. W. , van Hilten J. J. , Ducarmon Q. R. , Keller J. J. , Kuijper E. J. , and Contarino M. F. , Fecal Microbiota Transplantation in Neurological Disorders, Frontiers in Cellular and Infection Microbiology. (2020) 10, 10.3389/fcimb.2020.00098, 32266160. PMC7105733 32266160

[106] Xu H. M. , Huang H. L. , Zhou Y. L. , Zhao H. L. , Xu J. , Shou D. W. , Liu Y. D. , Zhou Y. J. , and Nie Y. Q. , Fecal Microbiota Transplantation: A New Therapeutic Attempt from the Gut to the Brain, Gastroenterology Research and Practice. (2021) 2021, 6699268, 10.1155/2021/6699268, 33510784. PMC7826222 33510784

[107] Kim D. Y. , Lee S. Y. , Lee J. Y. , Whon T. W. , Lee J. Y. , Jeon C. O. , and Bae J. W. , Gut Microbiome Therapy: Fecal Microbiota Transplantation vs Live Biotherapeutic Products, Gut Microbes. (2024) 16, no. 1, 2412376, 10.1080/19490976.2024.2412376, 39377231. PMC11469438 39377231

[108] Leeming E. R. , Johnson A. J. , Spector T. D. , and Le Roy C. I. , Effect of Diet on the Gut Microbiota: Rethinking Intervention Duration, Nutrients. (2019) 11, no. 12, 10.3390/nu11122862, 31766592. PMC6950569 31766592

[109] Shi N. , Nepal S. , Hoobler R. , Menni C. , Playdon M. C. , Spakowicz D. , Wells P. M. , Steves C. J. , Clinton S. K. , and Tabung F. K. , Pro-Inflammatory and Hyperinsulinaemic Dietary Patterns Are Associated With Specific Gut Microbiome Profiles: A TwinsUK Cohort Study, Gut Microbiome. (2024) 5, 10.1017/gmb.2024.14, 39703541. PMC11658949 39703541

[110] Kabisch S. , Hajir J. , Sukhobaevskaia V. , Weickert M. O. , and Pfeiffer A. F. H. , Impact of Dietary Fiber on Inflammation in Humans, International Journal of Molecular Sciences. (2025) 26, no. 5, 10.3390/ijms26052000, 40076626. PMC11900212 40076626

[111] Ma W. , Nguyen L. H. , Song M. , Wang D. D. , Franzosa E. A. , Cao Y. , Joshi A. , Drew D. A. , Mehta R. , Ivey K. L. , Strate L. L. , Giovannucci E. L. , Izard J. , Garrett W. , Rimm E. B. , Huttenhower C. , and Chan A. T. , Dietary Fiber Intake, the Gut Microbiome, and Chronic Systemic Inflammation in a Cohort of Adult Men, Genome Medicine. (2021) 13, no. 1, 10.1186/s13073-021-00921-y, 34140026. PMC8212460 34140026

[112] Limsrivilai J. , Sathirawich P. , Sattayalertyanyong O. , Pramyothin P. , Thaipisuttikul I. , Pausawasdi N. , Charatcharoenwitthaya P. , Sarasak R. , Nitayanon P. , Phaophu P. , Subdee N. , and Manatsathit S. , Increased Fiber Intake Enhances Clostridia Bacteria and Reduces Clinical Relapse in Patients With Clinically Quiescent Inflammatory Bowel Disease: A Prospective Longitudinal Study, JGH Open: An Open Access Journal of Gastroenterology and Hepatology. (2025) 9, no. 7, e70219, 10.1002/jgh3.70219, 40697428. PMC12280044 40697428

[113] Mu C. , Rho J. M. , and Shearer J. , The Interplay Between the Gut and Ketogenic Diets in Health and Disease, Advanced Science. (2025) 12, no. 36, e04249, 10.1002/advs.202504249, 40847749. PMC12463008 40847749

[114] Smolensky I. V. , Zajac-Bakri K. , Gass P. , and Inta D. , Ketogenic Diet for Mood Disorders From Animal Models to Clinical Application, Journal of Neural Transmission. (2023) 130, no. 9, 1195–1205, 10.1007/s00702-023-02620-x. PMC10460725 36943505

[115] Paredes-Marin A. , He Y. , and Zhang X. , Dietary Interventions in Metabolic Dysfunction-Associated Steatotic Liver Disease: A Narrative Review of Evidence, Mechanisms, and Translational Challenges, Nutrients. (2025) 17, no. 21, 10.3390/nu17213491, 41228562. PMC12608193 41228562

[116] Murillo-Cancho A. F. , Lozano-Paniagua D. , and Nievas-Soriano B. J. , Dietary and Pharmacological Modulation of Aging-Related Metabolic Pathways: Molecular Insights, Clinical Evidence, and a Translational Model, International Journal of Molecular Sciences. (2025) 26, no. 19, 10.3390/ijms26199643, 41096907. PMC12525316 41096907

[117] Marx W. , Lane M. , Hockey M. , Aslam H. , Berk M. , Walder K. , Borsini A. , Firth J. , Pariante C. M. , Berding K. , Cryan J. F. , Clarke G. , Craig J. M. , Su K. P. , Mischoulon D. , Gomez-Pinilla F. , Foster J. A. , Cani P. D. , Thuret S. , Staudacher H. M. , Sánchez-Villegas A. , Arshad H. , Akbaraly T. , O’Neil A. , Segasby T. , and Jacka F. N. , Diet and Depression: Exploring the Biological Mechanisms of Action, Molecular Psychiatry. (2021) 26, no. 1, 134–150, 10.1038/s41380-020-00925-x, 33144709. 33144709

[118] Pavlov V. A. and Tracey K. J. , The Vagus Nerve and the Inflammatory Reflex—Linking Immunity and Metabolism, Nature Reviews Endocrinology. (2012) 8, no. 12, 743–754, 10.1038/nrendo.2012.189, 23169440. PMC4082307 23169440

[119] Nemeroff C. B. , Mayberg H. S. , Krahl S. E. , McNamara J. , Frazer A. , Henry T. R. , George M. S. , Charney D. S. , and Brannan S. K. , VNS Therapy in Treatment-Resistant Depression: Clinical Evidence and Putative Neurobiological Mechanisms, Neuropsychopharmacology : Official Publication of the American College of Neuropsychopharmacology. (2006) 31, no. 7, 1345–1355, 10.1038/sj.npp.1301082, 16641939. 16641939

[120] Faraji N. , Payami B. , Ebadpour N. , and Gorji A. , Vagus Nerve Stimulation and Gut Microbiota Interactions: A Novel Therapeutic Avenue for Neuropsychiatric Disorders, Neuroscience and Biobehavioral Reviews. (2025) 169, 105990, 10.1016/j.neubiorev.2024.105990, 39716559. 39716559

[121] Thompson S. L. , O′Leary G. H. , Austelle C. W. , Gruber E. , Kahn A. T. , Manett A. J. , Short B. , and Badran B. W. , A Review of Parameter Settings for Invasive and Non-Invasive Vagus Nerve Stimulation (VNS) Applied in Neurological and Psychiatric Disorders, Frontiers in Neuroscience. (2021) 15, 709436, 10.3389/fnins.2021.709436, 34326720. PMC8313807 34326720

[122] de Melo P. S. , Gianlorenco A. C. , Marduy A. , Kim C. K. , Choi H. , Song J. J. , and Fregni F. , A Mechanistic Analysis of the Neural Modulation of the Inflammatory System Through Vagus Nerve Stimulation: A Systematic Review and Meta-Analysis, Neuromodulation : Journal of the International Neuromodulation Society. (2025) 28, no. 1, 43–53, 10.1016/j.neurom.2024.03.002, 38795094. 38795094

[123] Goggins E. , Mitani S. , and Tanaka S. , Clinical Perspectives on Vagus Nerve Stimulation: Present and Future, Clinical Science. (2022) 136, no. 9, 695–709, 10.1042/CS20210507. PMC9093220 35536161

[124] Ibrahim R. , Kambal A. , and Abdelmajeed M. A. , Glucagon-Like Peptide-1 Receptor Agonists in Neurodegenerative Diseases: A Comprehensive Review, Cureus. (2025) 17, no. 9, e92441, 10.7759/cureus.92441, 40964464. PMC12440237 40964464

[125] Nauck M. A. , Quast D. R. , Wefers J. , and Meier J. J. , GLP-1 Receptor Agonists in the Treatment of Type 2 Diabetes - State-of-the-Art, Molecular Metabolism. (2021) 46, 101102, 10.1016/j.molmet.2020.101102, 33068776. PMC8085572 33068776

[126] Du H. , Meng X. , Yao Y. , and Xu J. , The Mechanism and Efficacy of GLP-1 Receptor Agonists In the Treatment of Alzheimer′s Disease, Frontiers in Endocrinology. (2022) 13, 1033479, 10.3389/fendo.2022.1033479, 36465634. PMC9714676 36465634

[127] Dahiya D. and Nigam P. S. , Antibiotic-Therapy-Induced Gut Dysbiosis Affecting Gut Microbiota-Brain Axis and Cognition: Restoration by Intake of Probiotics and Synbiotics, International Journal of Molecular Sciences. (2023) 24, no. 4, 10.3390/ijms24043074, 36834485. PMC9959899 36834485

[128] Park J. C. , Chang L. , Kwon H. K. , and Im S. H. , Beyond the Gut: Decoding the Gut-Immune-Brain Axis in Health and Disease, Cellular & Molecular Immunology. (2025) 22, no. 11, 1287–1312, 10.1038/s41423-025-01333-3, 40804450. PMC12575876 40804450

[129] Daneman R. and Rescigno M. , The Gut Immune Barrier and the Blood-Brain Barrier: Are They so Different?, Immunity. (2009) 31, no. 5, 722–735, 10.1016/j.immuni.2009.09.012. 19836264

[130] Mayer M. G. and Fischer T. , Shared Mechanisms of Blood-Brain Barrier Dysfunction and Neuroinflammation in Coronavirus Disease 2019 and Alzheimer Disease, American Journal of Pathology. (2025) 195, no. 11, 2075–2087, 10.1016/j.ajpath.2025.03.011, 40254131. PMC12597690 40254131

[131] Mansour G. K. , Bolgova O. , Hajjar A. W. , and Mavrych V. , Neurovascular Dysfunction and Glymphatic Impairment: An Unexplored Therapeutic Frontier in Neurodegeneration, International Journal of Molecular Sciences. (2025) 26, no. 24, 11843, 10.3390/ijms262411843, 41465272. PMC12733005 41465272

[132] Cai Y. , Zhang Y. , Leng S. , Ma Y. , Jiang Q. , Wen Q. , Ju S. , and Hu J. , The Relationship Between Inflammation, Impaired Glymphatic System, and Neurodegenerative Disorders: A Vicious Cycle, Neurobiology of Disease. (2024) 192, 106426, 10.1016/j.nbd.2024.106426, 38331353. 38331353

[133] Guo H. , Tang X. , He X. , Weng Y. , Zhang Q. , Fang Q. , and Zhang L. , A Comprehensive Review of the Role of the Microbiota-Gut-Brain Axis via Neuroinflammation: Advances and Therapeutic Implications for Ischemic Stroke, Biomolecules. (2025) 15, no. 7, 10.3390/biom15070920, 40723792. PMC12292433 40723792

[134] Wenzel T. J. , Gates E. J. , Ranger A. L. , and Klegeris A. , Short-Chain Fatty Acids (SCFAs) Alone or in Combination Regulate Select Immune Functions of Microglia-Like Cells, Molecular and Cellular Neurosciences. (2020) 105, 103493, 10.1016/j.mcn.2020.103493. 32333962

[135] Ren H. and Liu Q. , Skull and Vertebral Bone Marrow in Central Nervous System Inflammation, Fundamental Research. (2024) 4, no. 2, 246–250, 10.1016/j.fmre.2023.01.012, 38933518. PMC11197483 38933518

[136] Di Vincenzo F. , Del Gaudio A. , Petito V. , Lopetuso L. R. , and Scaldaferri F. , Gut Microbiota, Intestinal Permeability, and Systemic Inflammation: A Narrative Review, Internal and Emergency Medicine. (2024) 19, no. 2, 275–293, 10.1007/s11739-023-03374-w, 37505311. PMC10954893 37505311

[137] Almansour N. , Al-Rashed F. , Choudhry K. , Alqaderi H. , Sindhu S. , Al-Mulla F. , and Ahmad R. , Gut Microbiota: A Promising New Target in Immune Tolerance, Frontiers in Immunology. (2025) 16, 1607388, 10.3389/fimmu.2025.1607388, 41050659. PMC12488628 41050659

[138] Wang S. , Sun S. , Liu H. , and Huang Q. , Research Progress in the Evaluation of Glymphatic System Function by the DTI-ALPS Method, Zhong nan da xue xue bao Yi xue ban = Journal of Central South University Medical sciences. (2023) 48, no. 8, 1260–1266, 10.11817/j.issn.1672-7347.2023.230091, 37875367. PMC10930843 37875367

[139] Rustenhoven J. , Pavlou G. , Storck S. E. , Dykstra T. , Du S. , Wan Z. , Quintero D. , Scallan J. P. , Smirnov I. , Kamm R. D. , and Kipnis J. , Age-Related Alterations in Meningeal Immunity Drive Impaired CNS Lymphatic Drainage, Journal of Experimental Medicine. (2023) 220, no. 7, 10.1084/jem.20221929, 37027179. PMC10083715 37027179

[140] Feng Y. , Wang S. , Xia H. , Jiang X. , Wu M. , Pan S. , and Song W. , Meningeal Lymphatics as a Therapeutic Target for Neurodegenerative Disorders, Translational Neurodegeneration. (2025) 14, no. 1, 10.1186/s40035-025-00528-y, 41366707. PMC12687538 41366707

[141] Li C. , Ren J. , Zhang M. , Wang H. , Yi F. , Wu J. , and Tang Y. , The Heterogeneity of Microglial Activation and Its Epigenetic and Non-Coding RNA Regulations in the Immunopathogenesis of Neurodegenerative Diseases, Cellular and Molecular Life Sciences : CMLS. (2022) 79, no. 10, 10.1007/s00018-022-04536-3, 36066650. PMC11803019 36066650

[142] Maurya S. K. , Gupta S. , and Mishra R. , Transcriptional and Epigenetic Regulation of Microglia in Maintenance of Brain Homeostasis and Neurodegeneration, Frontiers in Molecular Neuroscience. (2023) 15, 1072046, 10.3389/fnmol.2022.1072046. PMC9870594 36698776

[143] Lubomski M. , Xu X. , Holmes A. J. , Muller S. , Yang J. Y. H. , Davis R. L. , and Sue C. M. , The Gut Microbiome in Parkinson′s Disease: A Longitudinal Study of the Impacts on Disease Progression and the Use of Device-Assisted Therapies, Frontiers in Aging Neuroscience. (2022) 14, 875261, 10.3389/fnagi.2022.875261, 35656540. PMC9152137 35656540

[144] Mühlematter C. , Nielsen D. S. , Castro-Mejía J. L. , Walser J. C. , Schoch S. F. , and Kurth S. , Linking Gut Microbiota Rhythmicity to Circadian Maturation in Infants, Scientific Reports. (2025) 15, no. 1, 10.1038/s41598-025-25424-3, 41285943. PMC12644702 41285943

[145] Lorsch Z. S. and Liddle R. A. , Mechanisms and Clinical Implications of Gut-Brain Interactions, Journal of Clinical Investigation. (2026) 136, no. 1, 10.1172/JCI196346, 41480755. PMC12721889 41480755

[146] Solé-Guardia G. , Li H. , Willemse L. , Lebenberg J. , Jouvent E. , and Tuladhar A. M. , Imaging Brain Fluid Dynamics and Waste Clearance Involving Perivascular Spaces in Cerebral Small Vessel Disease, Alzheimer′s & Dementia: The Journal of the Alzheimer′s Association. (2025) 21, no. 4, e70212, 10.1002/alz.70212, 40289686. PMC12034940 40289686

[147] Chen F. , Heng T. , Feng Q. , Hua R. , Wu J. , Shi F. , Liao Z. , Qiao K. , Zhang Z. , and Miao J. , Quantitative Assessment of Brain Glymphatic Imaging Features Using Deep Learning-Based EPVS Segmentation and DTI-ALPS Analysis in Alzheimer′s Disease, Frontiers in Aging Neuroscience. (2025) 17, 1621106, 10.3389/fnagi.2025.1621106, 40741044. PMC12307369 40741044

[148] Londoño A. C. and Mora C. A. , High Efficacy Therapy to Prevent the Formation of Meningeal Tertiary Lymphoid Organs After CXCL13 Index Screening in Early Multiple Sclerosis, Frontiers in Neuroscience. (2025) 19, 1558810, 10.3389/fnins.2025.1558810, 40165834. PMC11955623 40165834

