# Supplementary material for: Barrier–Immune Gating Failure in Gut–Brain Neuroinflammation: A Working Hypothesis and Conceptual Framework
Source: Behav Neurol. 2026 Aug 20;2026:6222269. doi: 10.1155/bn/6222269 (PMC13493698; doi:10.1155/bn/6222269)
Supplement: Supplementary file 1 [file BN-2026-6222269-s001.docx]

**Supplementary Tables**

**Supplementary Table S1. Mechanistic checkpoints: evidence tiering, minimal readouts and confounders (Sections 2–4)**

| **Module (Section)** | **Evidence-tier boundary (Human/Animal/In vitro)** | **Decidable proposition (1 sentence)** | **Minimal readouts (recommended set)** | **Key confounders / alternative explanations** | **Translational levers (stratification/intervention)** |
| --- | --- | --- | --- | --- | --- |
| 2.1 TCVs / skull bone marrow–meninges axis (bone marrow priming) | Human: mostly correlational/proxy measures; Animal: trackable migration and temporal sequence; In vitro/ex vivo: lineage bias and chemotaxis/adhesion axes | Gut-derived low-grade inflammation can first reset the bone-marrow set point and amplify meningeal reactivity via near-field routes. | TCV flux (tracing/spatial readouts); meningeal myeloid composition; blood/CSF inflammation–hematopoiesis coupling | Systemic infection/drugs affecting hematopoiesis; global BBB disruption causing “pseudo-entry into brain” | Stratify by “bone marrow priming + meningeal reactivity”; target chemotaxis/adhesion axes or upstream barrier repair |
| 2.2 mLVs vs glymphatic system (clearance gating) | Human: imaging/fluid indices suggest clearance efficiency; Animal: causal tests by manipulating mLVs/AQP4; In vitro: lymphatic endothelium/astrocytic endfeet structural mechanisms | Reduced clearance rate can act as an upstream rate-limiting step, determining net protein accumulation and inflammatory gain. | Tracer-based clearance kinetics; AQP4 polarity; CSF turnover/drainage indices; PVS structural readouts | Sleep/anesthesia, vascular factors, baseline differences with ageing | Use “clearance rate” as an aligned endpoint; enhance drainage / improve sleep and vascular compliance |
| 2.3 BAMs (PVS gatekeepers: TMAO–CAA) | Human: metabolites/imaging correlate with CAA/microbleeds; Animal: validate metabolic input → BAM function → deposition; In vitro: lipid stress/lysosomal mechanisms | Peripheral metabolic load can reprogram perivascular clearance and inflammatory thresholds via BAMs, linking to vascular-wall pathology. | BAM state signatures; PVS clearance readouts; imaging endpoints for CAA/microbleeds; TMAO/choline metabolome profile | Metabolic comorbidities/medications; sampling site and disease-stage bias | Stratify by metabolic load + perivascular readouts; target metabolic pathways or enhance perivascular clearance |
| 3.1 SCFAs (HDAC / threshold modulation) | Human: mostly correlational; Animal: supplementation/depletion tests threshold shifts; In vitro: HDAC/acetylation and cell-type-specific effects | SCFAs act more like threshold modulators, reshaping glial response curves and homeostatic programs under the same stimulus. | SCFA levels; microglial homeostatic genes and response curves; barrier/IgA readouts | Diet composition, antibiotics, adherence, behavioral/circadian confounding | Align endpoints to “threshold readouts”; scalable upstream strategies using prebiotics/postbiotics |
| 3.2 Tryptophan–AhR axis (background setting) | Human: metabolome–phenotype associations; Animal: AhR manipulation tests persistence; In vitro: astrocyte/border-cell transcriptional programs | Indole–AhR sets an inhibitory baseline and persistence, determining whether inflammation crosses the threshold and becomes fixed. | AhR target-gene signatures; boundary-cell cytokine background; glial persistence readouts | Hepatic metabolism/drugs divert tryptophan flux; infection status | Stratify by “AhR signature + boundary background”; target metabolic shunting or receptor pathways |
| 3.3 Bile-acid axis and mitochondrial homeostasis | Human: bile-acid profiles correlate with phenotypes; Animal: receptor/pathway manipulation tests directionality; In vitro: organelle endpoints and accessibility | Bile acids provide an organelle-level mechanistic entry point, mapping peripheral metabolic signals onto mitochondrial homeostasis/stress endpoints. | Bile-acid profiles; ΔΨm/ROS/dynamics; inflammation–metabolism coupling readouts | Diet/hepatobiliary disease; alternative explanations from drug repurposing | Repurpose metabolic drugs and gut-targeted delivery; use organelle readouts as mechanistic endpoints |
| 3.4 Gut-derived peptides / vagal encoding (rapid tuning) | Human: abundant metabolic and some symptom endpoints; Animal: vagotomy/receptor-specific tests for directness; In vitro: receptor expression and signaling | Gut-derived peptides provide a high–time-resolution tuning layer that can rapidly reshape boundary gain and response amplitude. | Vagal activity/HRV; receptor-specific readouts; changes in boundary cytokine background | Indirect effects of metabolic improvement; sleep/medication confounding | Stratify by autonomic phenotype/boundary background; co-design with VNS and dietary timing |
| 4.1 DAM/BAMs state trajectories (replacing M1/M2) | Human: single-cell/spatial alignment to lesions; Animal: module-targeted interventions test cross-stage transitions; In vitro: lipid handling–lysosome–inflammation coupling | The key is the state threshold—whether it is crossed and yields sustained gain at boundary sites, rather than simple binary polarization. | Trajectory-stage markers; spatial localization (meninges/PVS/parenchyma); phagocytosis/lysosomal endpoints | Regional sampling bias; disease-stage confounding | Stratify by “trajectory stage + spatial niche”; target lipid-handling/lysosomal modules |
| 4.2 Meningeal T cells (boundary cytokine background) | Human: meningeal/CSF immune profiling associations; Animal: causal tests of cytokines; In vitro: direct effects on NVU/glia | The key question is not whether cells “enter the brain”, but whether a persistent boundary cytokine microenvironment forms. | Meningeal/CSF cytokines; excitability-related readouts; boundary cell composition | Peripheral infection; background immunosuppressive therapies | Use boundary cytokines for stratification and endpoints; combine barrier repair with clearance enhancement |

**Supplementary Table S2. Disease spectrum and translational strategies: evidence tiering, minimal readouts and confounders (Sections 5–6)**

| **Module (Section)** | **Evidence-tier boundary (Human/Animal/In vitro)** | **Decidable proposition (1 sentence)** | **Minimal readouts (recommended set)** | **Key confounders / alternative explanations** | **Translational levers (stratification/intervention)** |
| --- | --- | --- | --- | --- | --- |
| 5.1 Parkinson’s disease (flagship case) | Human: gut phenotypes correlate with staging; Animal: α-syn/inflammation models test the gating chain; In vitro: neuron–glia interactions | Gut-derived inputs are more likely to contribute to proteinopathy amplification via boundary gating and altered glial thresholds, rather than a single “direct-to-brain” route. | α-syn burden/spread; boundary ecology and clearance rate; peripheral immune set point | Stage heterogeneity; confounding by dopaminergic therapy | Stratify by “boundary gating + clearance”; prioritize trials aligned to mechanistic endpoints |
| 5.2 Alzheimer’s disease (flagship case) | Human: Aβ/tau relate to clearance/vascular factors; Animal: manipulate mLVs/AQP4/glia for causal tests; In vitro: phagocytosis–lysosome mechanisms | Clearance gating and perivascular gatekeepers can determine net protein accumulation and inflammatory amplification, explaining phenotypic heterogeneity. | Aβ/tau dynamics; clearance rate; CAA/microbleeds; glial trajectories | Vascular risk factors; sleep effects on tracer-based measures | Select participants by “clearance rate + perivascular readouts”; prioritize drainage enhancement/vascular strategies |
| 6.1 Live biotherapeutic interventions (LBT) | Human: heterogeneous efficacy and endpoints; Animal: mechanism and safety window; In vitro: strain function and metabolic outputs | Efficacy depends on aligning the dominant failed checkpoint with host context, rather than “generic replenishment”. | Strain functional outputs; barrier/immune set point; boundary readouts (CSF/imaging) | Antibiotics/diet/adherence; colonization failure | Stratify by “barrier/immune set point”; prioritize postbiotics/defined consortia |
| 6.2 FMT / postbiotic route | Human: constrained by donor effects and safety; Animal: donor–recipient matching and causality; In vitro: metabolite-combination effects | FMT is shifting from whole replacement toward specific functional modules/postbiotics to improve reproducibility and reduce risks. | Donor functional phenotypes; metabolite fingerprints; changes in recipient gating readouts | Infection risk; donor variability; regulatory constraints | Prioritize postbiotics/defined consortia; stratified trials bound to mechanistic readouts |
| 6.4 VNS / bioelectronic medicine | Human: parameter heterogeneity limits comparability; Animal: test targets and time windows; In vitro: neuro–immune nodes | The key is “parameter–population–endpoint alignment”, adjudicating efficacy with gating readouts rather than single symptom endpoints. | HRV/vagal activity; boundary cytokines; imaging/fluid mechanistic endpoints | Placebo effects; medication and sleep confounding | Stratify by autonomic phenotype; co-design with dietary timing/microbiome interventions |
| 6.5 Drug repurposing and gut-targeted strategies | Human: some drugs have RCTs but mechanisms are misaligned; Animal: disentangle direct vs indirect effects; In vitro: cell-type specificity | Drug value lies in converting gating checkpoints into controllable variables and verifying “target engagement” using mechanistic endpoints. | Mechanistic endpoints (clearance/boundary/threshold); PK–gut accessibility | Multi-target effects and alternative explanations via metabolic improvement | Bind trials to a “gating biomarker matrix” and move toward precision combination strategies |

**Abbreviations**

TCVs, trans-cortical vascular channels; mLVs, meningeal lymphatic vessels; PVS, perivascular spaces; BAMs, border-associated macrophages; DAM, disease-associated microglia; CAA, cerebral amyloid angiopathy; SCFAs, short-chain fatty acids; FMT, fecal microbiota transplantation; VNS, vagus nerve stimulation; HRV, heart rate variability; NVU, neurovascular unit.
