# Supplementary material for: Barrier–Immune Gating Failure in Gut–Brain Neuroinflammation: A Working Hypothesis and Conceptual Framework
Source: Behav Neurol. 2026 Aug 20;2026:6222269. doi: 10.1155/bn/6222269 (PMC13493698; doi:10.1155/bn/6222269)
Supplement: Supplementary file 2 [file BN-2026-6222269-s002.docx]

**Supplementary Table S3 (Extended). Additional disease-specific gating cues and minimal readouts (not expanded in the main text)**

| **Disease** | **Most relevant gating layer** | **Key cues (minimal readouts)** | **Why not expanded in the main text** |
| --- | --- | --- | --- |
| Parkinson’s disease (PD) | Propagation chain / proximal vagus–ENS gating | α-syn–related phenotypic stratification (body-first vs brain-first) plus gastrointestinal symptoms and RBD; when needed, pair with gut-derived inflammation and peripheral set-point readouts as comparators. | The evidence chain and controversies around “propagation” are extensive; addressing phenotyping and falsification routes would require a dedicated section and would dilute the manuscript’s primary adjudication framework centered on gating hierarchy. |
| Autism spectrum disorder (ASD) | Developmental-window threshold gating / synaptic pruning | Complement–synaptic pruning readouts (C1q/C3/CR3 axis) plus early-life inflammatory background; align behavioral dimensions to developmental time windows. | Exposure timing and the definition of developmental windows are core variables, and evaluation endpoints differ fundamentally from adult neurodegenerative spectra; a separate framework is needed. |
| Major depressive disorder (MDD) | Threshold resetting / immune–metabolic background setting | Inflammatory-phenotype stratification (hsCRP/IL-6/TNF-α) plus tryptophan–kynurenine pathway shifts; assess in parallel with sleep/circadian readouts. | Clinical endpoints are highly heterogeneous and strongly confounded by psychosocial variables and comorbidities; the main text therefore retains only the minimal chain that remains compatible with the gating-failure paradigm. |
