# Supplementary material for: Barrier–Immune Gating Failure in Gut–Brain Neuroinflammation: A Working Hypothesis and Conceptual Framework
Source: Behav Neurol. 2026 Aug 20;2026:6222269. doi: 10.1155/bn/6222269 (PMC13493698; doi:10.1155/bn/6222269)
Supplement: Supplementary file 3 [file BN-2026-6222269-s003.docx]

**Supplementary Table S4. Comparative strength, limitations, and alternative interpretations of evidence supporting the gating failure framework**

| **Evidence domain** | **Supportive evidence** | **Limiting or conflicting evidence** | **Alternative interpretation** |
| --- | --- | --- | --- |
| Skull bone marrow–meningeal axis | Animal tracing and anatomical evidence support skull-to-meninges immune supply | Human causal evidence remains limited | Systemic hematopoietic activation or vascular inflammation |
| Meningeal lymphatic/glymphatic clearance | Clearance impairment may align with protein accumulation | AQP4 and clearance are influenced by age, sleep, and vascular disease | Clearance failure may be secondary to neurodegeneration |
| SCFAs | Support barrier and epigenetic regulation in models | Human neurological outcomes are inconsistent | Conditional modulators, not universal anti-inflammatory mediators |
| Tryptophan–AhR | Supports astrocyte and immune-threshold regulation | Metabolites have divergent effects | Altered metabolism may be response rather than cause |
| BAMs/DAM | Cell-state models explain boundary and plaque responses | Human temporal evidence is limited | Glial remodeling may be disease-driven |
| AD | Clearance and microglial pathways fit the model | Dysbiosis may reflect diet, frailty, medication, or disease stage | Gut changes may be secondary |
| MS | Gut immune skewing may interact with Th17/Treg immunity | EBV, genetics, antigen specificity, and treatment effects may dominate | Microbiota may be a modifier rather than a primary trigger |
| Probiotics/FMT/VNS | Some mechanistic or symptomatic signals exist | Effects are inconsistent and endpoint-dependent | Host context and target engagement determine efficacy |
