# Supplementary material for: Barrier–Immune Gating Failure in Gut–Brain Neuroinflammation: A Working Hypothesis and Conceptual Framework
Source: Behav Neurol. 2026 Aug 20;2026:6222269. doi: 10.1155/bn/6222269 (PMC13493698; doi:10.1155/bn/6222269)
Supplement: Supplementary file 4 [file BN-2026-6222269-s004.doc]

**Scope and limitations of the barrier–immune gating failure hypothesis**

**Purpose of the model**

The barrier–immune gating failure framework is proposed as a working hypothesis to organize selected evidence linking intestinal signals, CNS boundary regulation, meningeal immunity, glial state transitions, and clearance dysfunction. It is intended to generate testable predictions rather than to serve as a universal explanation for all gut–brain interactions.

**What the model aims to explain**

The model is most applicable to contexts in which gut-derived microbial, metabolic, endocrine, or immune signals appear to be amplified at CNS interfaces, including the intestinal mucosal barrier, peripheral immune set points, skull bone marrow–meningeal communication, BBB/BCSFB regulation, meningeal immune niches, boundary-associated macrophages, microglial state transitions, and CSF–lymphatic or glymphatic clearance pathways.

**What the model does not claim**

This framework does not propose that neuroinflammation is universally linear, hierarchical, or barrier-dependent. It does not replace parallel gut–brain mechanisms, including vagal and autonomic signaling, neuroendocrine communication, microbial metabolite sensing, systemic immune activation, vascular responses, or direct metabolic effects. These pathways may operate simultaneously and may dominate in different diseases, stages, host backgrounds, or experimental models.

**When the model is most useful**

The framework is most useful when evidence shows measurable changes in CNS barrier permeability, meningeal immune organization, myeloid-cell trafficking, boundary-associated macrophage function, glial state transitions, AQP4 polarity, glymphatic or meningeal lymphatic clearance, or protein/inflammatory burden at CNS interfaces.

**Evidence needed for validation**

Stronger support requires temporal ordering, intervention reversibility, anatomical localization, cell-type-specific manipulation, and alignment between molecular, imaging, physiological, and functional endpoints. In the absence of these features, gut–brain associations should be interpreted cautiously and should not be assumed to represent gating failure.
